# The Role of Misshapen NCK-related kinase (MINK), a Novel Ste20 Family Kinase, in the IRES-Mediated Protein Translation of Human Enterovirus 71

**DOI:** 10.1371/journal.ppat.1004686

**Published:** 2015-03-06

**Authors:** Shi Yun Leong, Bryan Kit Teck Ong, Justin Jang Hann Chu

**Affiliations:** Laboratory of Molecular RNA Virology and Antiviral Strategies, Department of Microbiology, Yong Loo Lin School of Medicine, National University of Singapore, Singapore; University of California, Irvine, UNITED STATES

## Abstract

Human Enterovirus 71 (EV71) commonly causes Hand, Foot and Mouth Disease in young children, and occasional occurrences of neurological complications can be fatal. In this study, a high-throughput cell-based screening on the serine/threonine kinase siRNA library was performed to identify potential antiviral agents against EV71 replication. Among the hits, Misshapen/NIKs-related kinase (MINK) was selected for detailed analysis due to its strong inhibitory profile and novelty. In the investigation of the stage at which MINK is involved in EV71 replication, virus RNA transfection in MINK siRNA-treated cells continued to cause virus inhibition despite bypassing the normal entry pathway, suggesting its involvement at the post-entry stage. We have also shown that viral RNA and protein expression level was significantly reduced upon MINK silencing, suggesting its involvement in viral protein synthesis which feeds into viral RNA replication process. Through proteomic analysis and infection inhibition assay, we found that the activation of MINK was triggered by early replication events, instead of the binding and entry of the virus. Proteomic analysis on the activation profile of p38 Mitogen-activated Protein Kinase (MAPK) indicated that the phosphorylation of p38 MAPK was stimulated by EV71 infection upon MINK activation. Luciferase reporter assay further revealed that the translation efficiency of the EV71 internal ribosomal entry site (IRES) was reduced after blocking the MINK/p38 MAPK pathway. Further investigation on the effect of MINK silencing on heterogeneous nuclear ribonucleoprotein A1 (hnRNP A1) localisation demonstrated that cytoplasmic relocalisation of hnRNP A1 upon EV71 infection may be facilitated via the MINK/p38 MAPK pathway which then positively regulates the translation of viral RNA transcripts. These novel findings hence suggest that MINK plays a functional role in the IRES-mediated translation of EV71 viral RNA and may provide a potential target for the development of specific antiviral strategies against EV71 infection.

## Author Summary

Since its first isolation, human Enterovirus 71 (EV71) has been known to cause hand, foot and mouth disease in children, with some cases developing severe neurologic complications, leading to death. In the recent years, outbreaks within the Asia-Pacific region have caused significant deaths, making EV71 a major public health risk. Despite the growing threat from the spread of EV71 and increased research in this area, there are no clinically approved vaccines or antiviral drugs available against EV71. Cellular signalling and host kinases have been reported to play significant roles in the replication and propagation of many different pathogens. In this paper, we show that a serine/threonine kinase, Misshapen NIK-related kinase (MINK), plays a role in the replication of EV71 by stimulating the p38 mitogen activated protein kinase (p38-MAPK) pathway which in turns promotes the translation efficiency of EV71 viral protein synthesis. As the synthesis of viral proteins is crucial for the replication of the virus during infection, discovery of a crucial host kinase in this process may provide insights on the replication of EV71. With deeper understanding of the functions and regulation of MINK, this kinase may serve as a promising target for the development of antiviral therapy.

## Introduction

Human enterovirus 71 (EV71), a member of the *Picornaviridae* family and genus *Enterovirus*, is the major causative agent of hand-foot-and-mouth disease (HFMD). In recent years, EV71 has emerged as an important global health problem, causing significant deaths especially within the Asia-Pacific region [1]. Since its first isolation in 1969 in California [2], outbreaks have been observed worldwide, affecting countries such as Singapore, Malaysia and Taiwan [3,4,5,6]. EV71-associated HFMD often results in a higher risk of developing severe neurological complications and cardiopulmonary failure [7] which can be fatal. Despite the growing threat from the spread of EV71, there are no clinically approved vaccines or antiviral drugs available against EV71 to date [8] and treatments mainly aim to alleviate the symptoms [9].

EV71 is a small (33–35nm), single-stranded, positive-sense, non-enveloped RNA virus with a viral genome of approximately 7.5kb. The virions consist of an icosahedral capsid of 60 protomers surrounding viral genomic RNA [10] that contains a single open reading frame (ORF) flanked by the 5’ untranslated region (UTR) and the 3’ UTR. The ORF encodes four structural proteins (VP1, VP2, VP3 and VP4) that make up the viral capsid and seven non-structural proteins (2A, 2B, 2C, 3A, 3B, 3C and 3D) which are involved in viral replication. Viral 2A and 3C proteases are involved in the cleavage of the polyprotein precursor to release the mature viral proteins while viral protein 3D is the RNA-dependent RNA polymerase (RdRp) that plays a major role in the synthesis of negative- and positive-sense viral RNA [11]. Upon EV71 infection, the viral genome is translated into the viral polyprotein and the 3D protein participates in the transcription of the positive-sense genomic viral RNA into the complementary negative-sense viral RNA, which serves as a template for the synthesis of more positive-sense genomic viral RNA. The genomic RNA is then translated into more viral polyproteins in a cap-independent manner and the polyproteins are subsequently processed into the structural capsid proteins and non-structural proteins [11]. The 5’ UTR of the EV71 genomic RNA contains a cloverleaf structure involved in viral RNA replication and an internal ribosomal entry site (IRES) which directs viral protein translation in a cap-independent manner [12,13]. The picornavirus IRES have been classified into three types based on its structure and enteroviruses (and rhinoviruses) have the type 1 IRES which requires certain eukaryotic initiation factors (eIFs) and IRES-specific transacting factors (ITAFs) to initiate viral protein translation. In contrast to cellular cap-dependent translation, the host 40S ribosomal subunit is recruited at the IRES without the need for eIF4E to initiate viral polyprotein translation [14]. A number of ITAFs have been identified to interact with picornavirus IRES and mediate translation initiation of the viral polyprotein. These ITAFs include polypyrimidine tract-binding protein (PTB) [15,16,17], heterogeneous nuclear ribonucleoprotein E (hnRNP E) [18], far-upstream element-binding protein 1 (FBP1) and FBP2 [19]. Among these ITAFs, hnRNP A1[20],and FBPs [19] have been reported to interact with EV71 IRES.

Given the small genome size of EV71, the virus depends on several cellular proteins and machineries in the host cell to complete its replication. In search for host cellular factors that play a role in EV71 replication, an understanding of the cellular proteins involved in different stages of viral replication would be useful for the identification of potential targets for the development of antiviral strategies. In this study, an immunofluorescence cell-based virus infection assay was set-up to screen the human serine/threonine kinase siRNA library using a set of validated small interfering RNAs (siRNAs) targeting the host serine/threonine kinases. Several candidate kinases that showed significant inhibition of EV71 replication upon gene knockdown were identified and among the hits, Misshapen/NIKs-related kinase (MINK), a novel sterile 20 (Ste20) family kinase, was chosen for further evaluations. MINK, also known as MAP4K6, is a germinal center kinase (GCK) from the Ste20 family of kinases that includes more than 30 serine/threonine kinases with catalytic domains that are homologous to the yeast Ste20 kinases. MINK is structurally similar to the Nck-interacting kinase (NIK) which has previously been proposed to link the protein tyrosine kinase signals to the activation of c-Jun N-terminus kinases (JNK) pathway via the SH2-SH3 domain of Nck [21]. As a member of the GCK class of kinases, MINK has an N-terminal kinase domain and a C-terminal regulatory domain. The intermediate domain consists of multiple proline rich motifs that are putative SRC homology 3 (SH3) binding sites [22]. The MINK1 gene encodes a polypeptide of 1312 amino acids and is expressed in most tissues in at least five alternatively spliced isoforms [22]. Studies on cells under environmental stress [23] revealed that MINK activates the JNK and p38 MAPK pathway, which are important signalling pathways involved in various cellular functions such as apoptosis, protein translation and cell differentiation [24]. Apart from cellular functions, p38 MAPK pathway has also been reported to play a role in the IRES-mediated viral protein translation of Encephalomyocarditis virus (EMCV) viral RNA [25]. In this study, we revealed the involvement of MINK in EV71 replication and further elucidated the mechanism through which MINK regulates the synthesis of EV71 viral polyprotein upon viral infection.

## Results

### Development of a screening assay for EV71 replication based on indirect immunofluorescence

A screening assay was previously developed to screen for host factors involved in EV71 replication using targeting siRNA [26]. In this screen, a similar screening approach was adopted based on immunofluorescence assay to detect EV71 structural protein expression as an indicator of successful EV71 infection and replication. Positive control wells containing EV71-infected cells without siRNA treatment had a mean of 51.600% antigen positive cells with a standard deviation of 5.407%. Mock-infected cells were used as negative controls to verify the specificity of the antibody. In addition, the data were analysed by applying Z-score statistics and a Z’ factor of 0.673 was obtained from the primary screen, indicating that the screening platform was sufficiently robust and was suitable for the high-throughput screening of the human serine/threonine kinase siRNA library.

### MINK plays an essential role in EV71 replication

**Fig 1:**
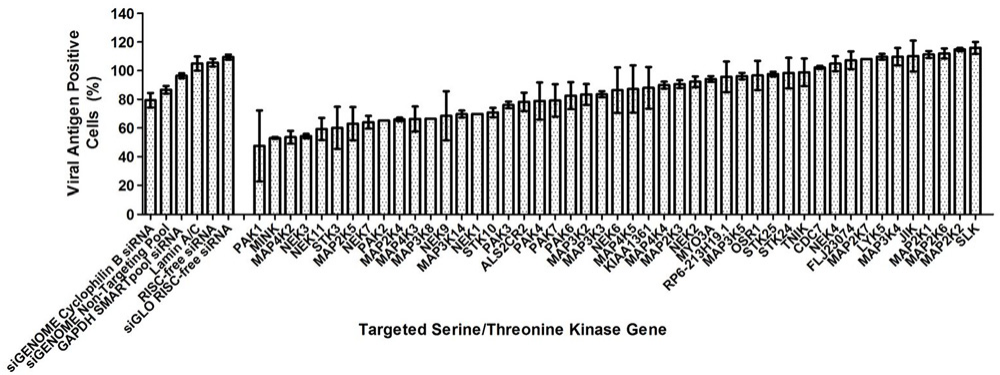
Human serine/threonine kinase siRNA library screen. Effect of gene knockdown by siRNA on EV71 replication analysed from primary screen. 40% reduction in viral antigen positive cells was considered as the acceptable level of virus inhibition and positive hits are genes which resulted in a percentage of viral antigen positive cells of less than 60% upon the knockdown of these genes. As such, 6 genes have been identified as positive hits from the primary screen. First 6 bars represent siRNA controls used while the other bars represent the host serine/threonine kinases targeted in the screening. siRNA controls utilised included non-targeting siRNAs as well as siRNAs targeting several housekeeping genes. Values obtained in the graph were normalised against the mean of the transfection control (EV71-infected cells treated with only the transfection reagent).

A human serine/threonine kinase siRNA library that targets 47 serine/threonine kinases (S1 Table) was utilised in the primary screen in search for human serine/threonine kinases involved in EV71 replication. Using the criteria of ≥40% inhibition to identify positive hits in the primary screen, 6 serine/threonine kinases were identified as positive hits (Fig. 1). The top three targets identified, PAK1, MINK and MAP4K2, were first analysed. PAK1 was observed to cause the highest level of virus inhibition but closer analysis led to the removal of this target from further downstream studies due in part to the large standard error in the results obtained and lower cell density observed in all the PAK1 replicate wells. MINK and MAP4K2 were identified as putative targets crucial to EV71 replication and validation of their involvement was carried out using both immunofluorescence assay and viral plaque assay.

**Fig 2:**
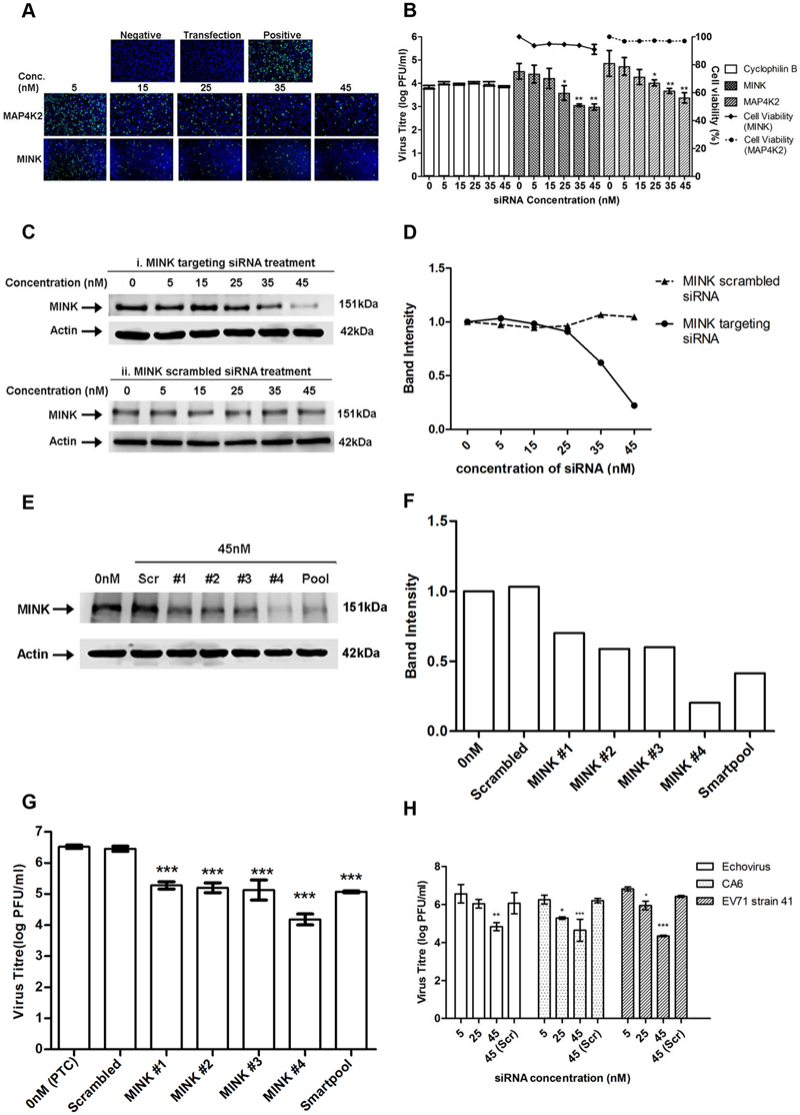
Silencing of MINK significantly reduced EV71 replication in a siRNA concentration-dependent manner. (A) siRNA-treated EV71 infected cells were fixed at the same time-points and intracellular viruses were detected by immunofluorescence assay. Immunofluorescence detection of EV71 VP2 proteins (green) with the nuclei stained with DAPI (blue) is shown. The images were taken at 10X magnification. Cells in both the negative and transfection controls were not infected with EV71 while cells in the positive control were infected with EV71 in the absence of siRNA. (B) Cell viability of siRNA-treated cells was measured in relation to untreated cells using alamarBlue assay after 72h incubation. Virus titres in the supernatant of siRNA-treated cells were analysed via viral plaque assay. Error bars represent standard deviation (SD) of triplicate data and values obtained were normalised against the transfection control. Statistical analyses were performed using one-way ANOVA and Dunnett’s test (Graphpad software) against untreated control. *P <0.05 (n = 3), **P <0.01 (n = 3). (C) Verification of gene knockdown efficiency of MINK siRNA SMARTpool at concentrations ranging from 0nM to 45nM. Western blot analysis was performed to detect protein expression levels of MINK, with β-actin as the loading control. Parallel transfection of scrambled siRNA served as a knockdown control. MINK protein expression was observed to decrease in a dose-dependent manner across siRNA concentration. (D) Band intensity of MINK gene knockdown verification. The band intensities representing MINK protein expression level were quantitated with reference to actin control bands (for each individual concentration) and PTC. The intensities of protein bands were quantitated using ImageJ Gel Analysis program. (E) Verification of gene knockdown efficiency of individual siRNA within the siRNA SMARTpool directed against MINK at 45nM. Western blot analysis was performed to detect protein expression levels of MINK, with β-actin as the loading control. (F) Band intensity of MINK gene knockdown verification in deconvolution assay. The band intensities representing MINK protein expression level were quantitated with reference to actin control bands (for each individual siRNA) and PTC. (G) Virus titres in the supernatant of cells treated with individual siRNAs within siRNA SMARTpool were analysed via viral plaque assay. Error bars represent standard deviation (SD) of triplicate data. Statistical analyses were performed using one-way ANOVA and Dunnett’s test (Graphpad software) against untreated control. ***P <0.0001 (n = 3). (H) Virus titres of other human enteroviruses (Echovirus 7, Coxsackievirus A6 and EV71 strain 41) in the supernatant of siRNA-treated cells were analysed via viral plaque assay. Error bars represent standard deviation (SD) of triplicate data. Statistical analyses were performed using one-way ANOVA and Dunnett’s test (Graphpad software) against scrambled control (Scr). *P < 0.05, **P < 0.01 and ***P < 0.0001 (n = 3) versus scrambled control.

Increasing concentrations of siRNA resulted in a reduction of immunofluorescence-detectable EV71 replication for cells treated with siRNA against either MINK or MAP4K2 (Fig. 2A). This coincided with the dose-dependent reductions up to 1.5 log for both siRNAs at 45nM in infectious virus released from the cells as indicated by viral plaque assays (Fig. 2B). As the siRNA targeting cyclophilin B has been utilised previously in the primary screen as an siRNA control, it was also included in the secondary assay to ensure that the effect of infectious virus titre reduction was not due to off-target effects. Furthermore, minimal cellular cytotoxicity was observed across the concentrations of both MINK and MAP4K2 siRNAs (Fig. 2B), indicating that the inhibition of EV71 replication was not due to the cytotoxic effects of the siRNAs at the range of concentrations used.

In view of the higher levels of virus inhibition upon the silencing of MINK and its unknown function in virus replication, MINK was selected for further investigation. Western blotting was carried out to verify the gene knockdown efficiency. Dose-dependent reduction in the protein expression level of MINK was observed upon MINK siRNA treatment, suggesting that the range of siRNA concentration used in this study was effective in silencing the MINK gene (Fig. 2C panel i and Fig. 2D). This was further verified by the scrambled siRNA treatment, as there was no reduction in MINK protein levels observed across the siRNA concentration range used in the study (Fig. 2C panel ii, and Fig. 2D). To further validate the specificity of the MINK SMARTpool siRNAs, deconvolution assay was performed with 45nM concentrations of each specific individual siRNA of the SMARTpool (four specific siRNAs). This approach would help to ensure that inhibitory effects of the targeting siRNAs on EV71 infection observed in the secondary assay was specific and not due to off-target gene effects. Before a viral plaque assay was performed to determine the virus titre upon gene knockdown using the individual siRNAs within the SMARTpool, gene knockdown efficiency of each individual siRNAs was assessed. From the Western blot analysis, it was observed that all four individual siRNAs directed against MINK were effective in reducing MINK protein levels at a concentration of 45nM (Fig. 2E and Fig. 2F). Coinciding with the knockdown efficiency of individual siRNA, viral plaque assay results showed that all four individual siRNAs resulted in significant reduction in virus titre of at least 1.3 log at 45nM concentration (Fig. 2G). Taken together, these findings demonstrated that the inhibition on EV71 propagation was a result of the targeted siRNA knockdown of MINK and thus, MINK is essential for the replication of EV71.

### MINK is essential for the replication of other human enteroviruses

To investigate if MINK plays a role in other human enteroviruses as well, MINK siRNA-treated cells were infected with various human enteroviruses: a different strain of EV71 (EV71 strain 41), Coxsackievirus A6 (CA6) and Echovirus 7 at MOI 1. The dose-dependent reduction in infectious virus titres upon the siRNA knockdown of MINK was reproduced with all three viruses as demonstrated in Fig. 2H. As shown in Fig. 2H, the knockdown of MINK resulted in the reduction of infectious virus titre by approximately 2.3 log units. 1.5 log unit and 1.5 log unit respectively for EV71 strain 41, Echovirus 7 and CA6 relative to the scrambled siRNA controls (Scr). This suggested that the involvement of MINK is not restricted to EV71, but may extend to other human enteroviruses as well.

### Silencing of MINK does not affect EV71 entry

**Fig 3:**
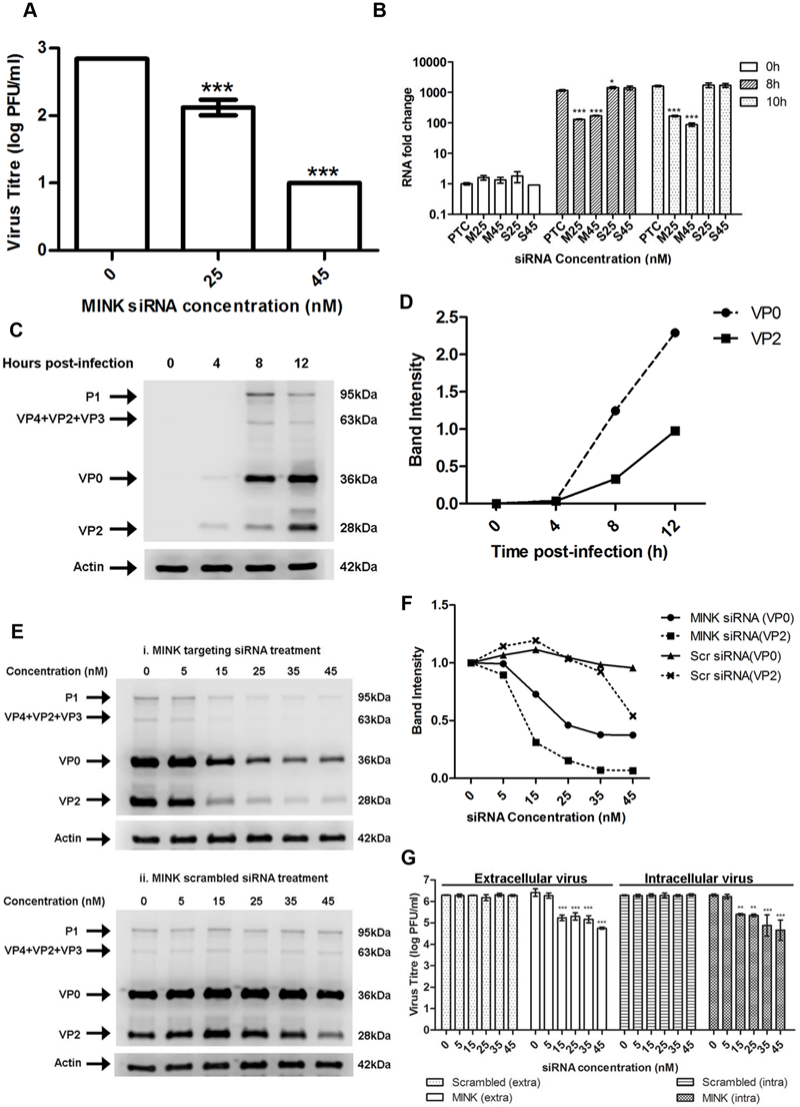
MINK plays an essential role in EV71 viral protein synthesis. (A) EV71 viral RNA was transfected into RD cells pre-treated with MINK siRNA and supernatant was harvested from cells at 12h post-infection (hpi) for viral plaque assay. Silencing of MINK with targeting siRNA continued to cause inhibition of virus replication. Statistical analysis was performed using one-way ANOVA with Dunnett’s test (Graphpad software). *** *P* < 0.0001 (n = 3) versus untreated control (0nM). (B) EV71 RNA synthesis was sensitive to silencing efficiencies of MINK. Quantitative RT-PCR assay revealed significant reduction in levels of EV71 RNA across increasing siRNA concentration in MINK siRNA-treated cells. Total RNA was extracted for all samples at 0, 8 and 10hpi and EV71 RNA levels were measured. C_T_ values were normalised against actin and relative quantification of viral RNA level was determined. The ΔΔCt data were calculated from three independent experiments and error bars represent standard deviation for triplicate data sets. Fold difference of viral RNA for all samples was calculated relative to the RNA level in the transfection control (PTC) at 0hpi. Statistical analyses were carried out using one-way ANOVA with Dunnett’s test (Graphpad software). *P<0.05 and *** *P* < 0.0001 (n = 3) vs the respective PTC at each time-point. (C) Time course study of EV71 structural protein expression via Western blot analysis. Upper band (36kDa) represents VP0 while lower band (28 kDa) represents VP2. β-actin was used as the loading control. (D) Band intensity of VP0 and VP2 in time course study. The band intensities representing VP0 and VP2 protein expression level were quantitated with reference to actin control bands (for each time-point) and 0hpi using ImageJ Gel Analysis program. (E) Viral protein expression levels upon the silencing of MINK. VP0 and VP2 viral protein expression was observed to decrease with increasing concentration of siRNA targeting MINK. (F) Band intensities of VP0 and VP2 upon siRNA knockdown of MINK. The band intensities representing VP0 and VP2 protein expression level were quantitated with reference to actin control bands (for each siRNA concentration) and 0nM using ImageJ Gel Analysis program. (G) Extracellular and Intracellular virion levels upon the silencing of MINK. Extracellular EV71 virions in the supernatant and intracellular virus particles were harvested separately at 12hpi for viral plaque assay to assess the effect of siRNA knockdown of MINK on virus packaging and release. Silencing of MINK resulted in significant reduction in both intracellular and extracellular virions. Statistical analysis was performed using one-way ANOVA with Dunnett’s test (Graphpad software). **P < 0.01 and *** *P* < 0.0001 (n = 3) versus untreated control (0nM).

Further experiments were performed to elucidate the involvement of MINK within the different stages of the EV71 replication processes (viral entry, viral RNA replication and viral protein synthesis). To assess the involvement of MINK in viral entry, viral RNA was extracted and transfected into cells which were pre-treated with MINK siRNA to bypass the normal viral entry processes ie. clathrin-mediated endocytosis for EV71 [26]. As such, infectious virus titre obtained from viral plaque assays would assist in the elucidation of the potential involvement of MINK in the viral entry stage. In this assay, a dose-dependent reduction in the virus yield was observed across the siRNA concentrations with a maximum reduction of ~1.8 log at 45nM (Fig. 3A), indicating that the silencing of MINK continued to cause virus inhibition despite bypassing the viral entry stage. This result suggested that MINK might play a more essential role at the post-entry stage.

### MINK plays an essential role in EV71 viral protein synthesis and viral RNA synthesis

To examine the involvement of MINK in the post-entry stages of EV71 replication, viral RNA synthesis of EV71 was determined by quantitative RT-PCR on viral RNA samples extracted from infected RD cells pre-treated with either MINK siRNA (M) or scrambled siRNA (S) control at 25nM (M25 and S25) and 45nM (M45 and S45). A background control measuring the viral RNA levels at 0h post-infection was included to account for the background viral RNA resulted from virus entry and the binding of residual virions on the cell surfaces. The infected cells pre-treated with the siRNA were harvested at 8h and 10h post-infection to examine the relative amount of viral RNA. Fold change in RNA level for all samples was calculated relative to the RNA level in the transfection control (PTC/ 0nM) at 0h post-infection. Comparison of RNA level was made between the samples treated with MINK siRNA (M25 and M45) or scrambled siRNA (S25 and S45) and their PTC at each time-point. Results showed increase in the level of viral RNA at 8h and 10h post-infection, relative to 0h background control, indicating that there was viral RNA replication upon siRNA treatment. The viral RNA level in MINK siRNA-treated cells was significantly lower than that in the transfection control (PTC) at both 8h and 10h post-infection (Fig. 3B), with fold reductions of 6.8 and 18.4 at 8h and 10h post-infection at 45nM concentration, respectively. On the contrary, there was no significant change in the viral RNA level in the samples treated with the scrambled siRNA control at both concentrations compared to PTC. These results indicated that the silencing of MINK has inhibitory effects on the production of viral RNA.

Since the production of viral proteins precedes and is essential for the synthesis of viral RNA, the influence of MINK silencing on EV71 replication at the level of translation was determined. A time course study was first conducted to identify the time period during which EV71 viral protein expression occurs (Fig. 3C and 3D). Across a time course of 12h after EV71 infection, greatest increase in the EV71 structural viral protein expression, VP0 (~36kDa) and VP2 (~28kDa), was observed between 4 and 8h post-infection (Fig. 3C and 3D). As such, 8h post-infection was selected as the time-point for further analysis on the role of MINK in viral protein synthesis. At 8h post-infection, a dose-dependent reduction in the VP0 and VP2 was observed in EV71-infected cells pre-treated with MINK siRNA (Fig. 3E panel i and 3F) as opposed to the constant protein expression level of VP0 and VP2 in cells treated with scrambled MINK siRNA (Fig. 3E panel ii and 3F). Similarly, reduction in non-structural protein level such as the 3D protein (RNA-dependent RNA polymerase) was observed in cells pre-treated with MINK siRNA but not in cells treated with scrambled siRNA (S2A-C Fig). The reduction in the 3D protein level also corresponded with the reduction in the viral RNA level. Since the depletion of MINK protein in cells led to a corresponding reduction in the viral proteins synthesised, MINK is likely to be involved in the stage of viral protein synthesis.

### Silencing of MINK does not block virus release

To further confirm the stage of involvement of MINK in the replication of EV71, intracellular and extracellular EV71 virions were quantified at 12h post-infection to determine whether MINK plays a role in viral packaging and release. Although a significant reduction of ~1.5 log was observed in the extracellular virions upon the siRNA knockdown of MINK at 45nM, a significant reduction in virus titre was also observed in the intracellular virions (Fig. 3G). Hence, we concluded that the reduction in the amount of virus released was not due to a blockage in the virus release process upon the silencing of MINK, but was due to a decrease in the total production of virus particles.

### Replication of EV71 triggers MINK phosphorylation

**Fig 4:**
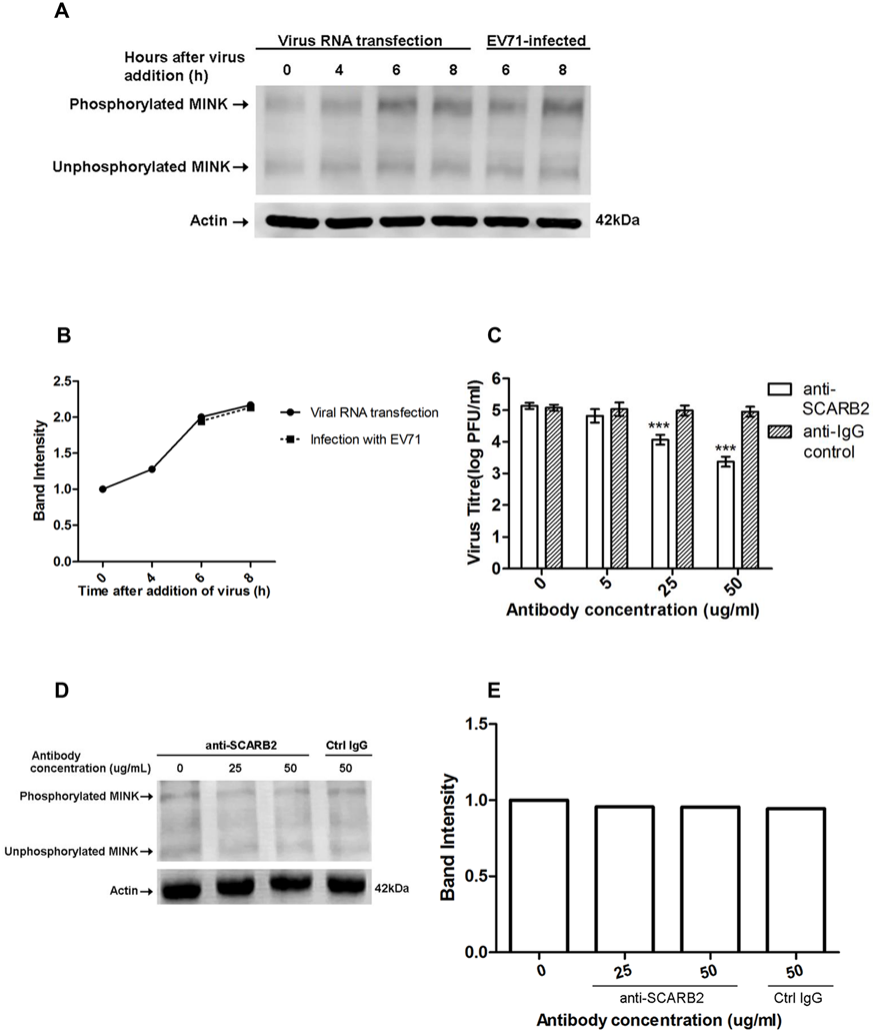
Phosphorylation of MINK is triggered post-entry by early replication events. Phos-tag acrylamide binds phosphorylated proteins and retards their migration to separate the phosphorylated proteins from their unphosphorylated counterparts. Total MINK antibody was used to detect both phosphorylated (upper bands) and unphosphorylated MINK (lower bands). β-actin was used as a loading control. (A) Viral RNA was transfected into cells and cell lysates were harvested at indicated time-points to assess the phospho-MINK levels. Phospho-MINK levels in RNA-transfected cells were comparable to the infection control at the same time-points. (B) The band intensities representing MINK phosphorylation level were quantitated with reference to actin control bands (for each time-point) and 0h using ImageJ Gel Analysis program. (C) Virus titres in the supernatant of cells treated with the anti-SCARB2 and anti-IgG antibodies were analysed via viral plaque assay. Blocking SCARB2 receptors with increasing concentration of SCARB2 antibody resulted in a significant reduction in virus titres. Error bars represent standard deviation (SD) of triplicate data. Statistical analyses were performed using one-way ANOVA and Dunnett’s test (Graphpad software) against untreated control. ***P <0.0001 (n = 3) (D) Blocking SCARB2 receptors with increasing concentration of SCARB2 antibody did not affect the phosphorylation of MINK in cells at 6h after addition of virus. (E) The band intensities representing MINK phosphorylation level were quantitated with reference to actin control bands (for each concentration) and 0μg/mL using ImageJ Gel Analysis program.

As a MAP kinase kinase kinase kinase (MAP4K), MINK is activated upstream in MAPK pathways and thus we hypothesised that the early events in EV71 infection could be responsible for the activation of MINK. To investigate if virus binding and entry triggered the phosphorylation of MINK, Western blot analysis on phospho-MINK was conducted after the transfection of viral RNA into cells to bypass the normal entry processes of EV71. Since phospho-MINK antibodies are not available commercially, a phosphate-binding tag (Phos-Tag) [27] was used to separate the phosphorylated proteins from the unphosphorylated proteins. 6h and 8h were selected as the time-points for harvest of cell lysates due to the significant increase in phospho-MINK levels at these time-points after infection (S1 Fig). As shown in Fig. 4A and Fig. 4B, cells transfected with viral RNA displayed similar phospho-MINK levels at 6h and 8h as the infection control (EV71-infected), suggesting that initial binding and entry processes of the virus was not required for the activation of MINK upon EV71 infection.

To further ascertain that initial binding of the virus was not required for the activation of MINK, an infection inhibition assay was performed. It has been well-established that scavenger receptor class B2 (SCARB2) is the cellular receptor for EV71 on RD cells [28], hence an antibody to SCARB2 was used to block the cellular SCARB2 receptors to prevent virus binding. Viral plaque assay was first conducted to confirm the efficacy of the anti-SCARB2 antibody in blocking EV71 infection. A corresponding control IgG antibody that does not bind specifically to any proteins was used as a negative control. Results from the infection inhibition assay showed that pre-treatment of RD cells with the anti-SCARB2 antibody blocked EV71 infection in a dose-dependent manner as the virus titre was significantly reduced by ~1.5 log at the highest concentration of anti-SCARB2 antibody used (Fig. 4C). On the other hand, treatment with the control IgG antibody did not affect the binding and entry of virus into the cells (Fig. 4C) as the virus titres remained constant across increasing concentrations of IgG antibody.

To investigate if virus binding to cellular SCARB2 triggered the activation of MINK, EV71-infected RD cells pre-treated with the anti-SCARB2 antibody were lysed at 6h after addition of virus for Western blot analysis. Our results indicated that inhibition of the virus binding to SCARB2 with increasing concentration of the antibody did not reduce the phospho-MINK levels in the cells as the phospho-MINK levels in cells treated with 25 and 50μg/mL of anti-SCARB2 antibody showed similar level as that in cells treated with 50μg/mL of control IgG antibody (Fig. 4D and 4E). As such, virus binding was unlikely to be the triggering event of the phosphorylation of MINK. Together, the activation profile of MINK (S1 Fig) and the entry assays suggested that the phosphorylation of MINK was stimulated post-entry, in the early phase of viral replication which occurs during the 6h period after addition of virus.

### EV71 infection activates p38 MAPK in RD cells

**Fig 5:**
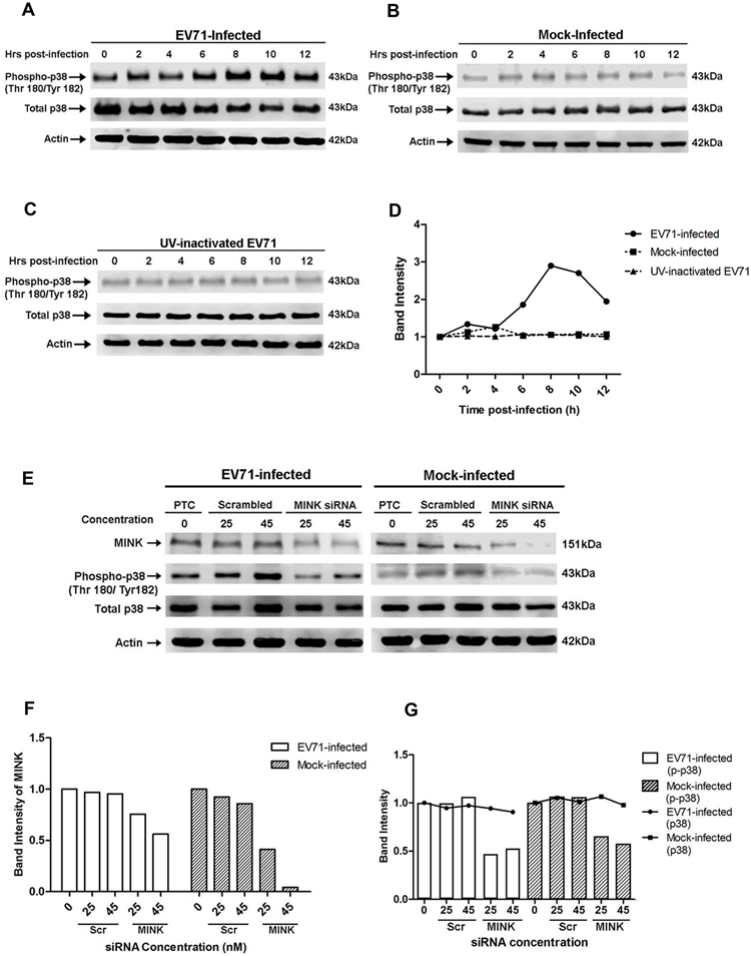
EV71 infection triggers p38 MAPK phosphorylation downstream of MINK. Western blot analysis was performed to assess the levels of phosphorylated p38 MAPK (phospho-p38) at 0, 2, 4, 6, 8, 10 and 12hpi. Total p38 (t-p38) was probed as an internal control for p38 MAPK protein expression and β-actin was used as a loading control. (A) Infection with infectious EV71 was observed to activate p38 MAPK phosphorylation from 6hpi and was most significant at 8hpi. (B) Phosphorylation levels of p38 MAPK in mock-infected cells was basal and constant across the 12h. (C) Cells exposed to UV-inactivated EV71 virus also displayed basal and constant level of p38 MAPK phosphorylation of p38 MAPK. (D) Quantification of phospho-p38 MAPK (Thr180/Tyr182) protein bands. The band intensities representing phospho-p38 MAPK level were quantitated with reference to actin control bands (for each time-point) and 0hpi using ImageJ Gel Analysis program. (E) Western blot analysis of the phosphorylation levels of p38 MAPK at 8hpi in siRNA-treated cells. The left panel shows the phospho-p38 levels in EV71-infected under three different treatments: no treatment, scrambled siRNA treatment and MINK targeting siRNA treatment. The right panel shows the phospho-p38 levels in mock-infected cells under the same treatments. (F) Quantification of MINK protein bands with reference to actin control bands (for each concentration) and PTC using ImageJ Gel Analysis program. (G) Quantification of phospho-p38 MAPK (Thr180/Tyr182) and total p38 protein bands with reference to actin control bands (for each concentration) and PTC using ImageJ Gel Analysis program.

After determining the triggering event of MINK upon EV71 infection, we next investigated the mechanism of action of MINK on EV71 viral protein synthesis. It has been reported that MINK activates the p38 MAPK pathway [23], a signalling pathway that has also been shown to play a role in the replication of Encephalomyelitis virus (EMCV), a member of the *Picornaviridae* family [25]. As such, we examined the activation profile of p38 MAPK upon EV71 infection to assess whether the p38 MAPK signalling pathway is activated during EV71 replication. As serum has also been reported to induce phosphorylation of certain proteins [29], fecal calf serum (FCS) was removed from the virus stock and growth media in the course of this experiment to reduce the additional activation of p38 MAPK by the serum. Cell lysates were analysed at indicated time-points post-infection for 12h by Western blotting to examine the changes in the phosphorylation levels of p38 MAPK (phospho-p38). Constant and basal phosphorylation of p38 MAPK was observed in mock-infected cells throughout the 12h time course (Fig. 5B and 5D). In contrast, the EV71-infected cells showed an increase in the phosphorylation level of p38 MAPK between 6 to 8h post-infection (Fig. 5A and 5D), followed by a subsequent decrease from 8 to 12h post-infection. To demonstrate the dependency of p38 MAPK phosphorylation on EV71 replication, we also examined the phospho-p38 MAPK profile in RD cells infected with UV-inactivated EV71 (Fig. 5C and 5D). Similar to the mock-infected control, cells exposed to UV-inactivated EV71 showed constant phosphorylation level of p38 MAPK throughout the 12h time course, indicating that attachment of the virions to cell surface receptors or virus entry process were not sufficient to trigger the phosphorylation of p38 MAPK. Total p38 MAPK was also assessed to ensure that the changes in phospho-p38 MAPK levels were not due to differences in p38 MAPK expression levels. Phospho-p38 MAPK levels at 0h post-infection appears higher in Fig. 5A in EV71-infected samples than that in the mock-infected samples (Fig. 5B) and samples exposed to UV-inactivated EV71 (Fig. 5C) probably due to more total proteins loaded as seen from the total p38 and β-actin levels. However, it is evident in the trend of p38 MAPK activation profile that phospho-p38 MAPK levels were significantly increased upon EV71 infection (Fig. 5D). Hence, these results suggested that activation of p38 MAPK signalling pathway requires the active replication of EV71.

### MINK contributes to the activation of p38 MAPK during EV71 infection

To establish a link between the activation of MINK and p38 MAPK phosphorylation upon viral infection, cells were pre-treated with either scrambled MINK or MINK siRNA prior to EV71 infection and lysed at 8h post-infection for Western blot analysis. Efficacy of the MINK siRNA was demonstrated with a dose-dependent reduction in MINK protein levels upon knockdown (Fig. 5E and 5F). Dose-dependent reduction in phospho-p38 MAPK protein levels was also observed with increasing MINK siRNA concentration (25nM and 45nM) in both EV71-infected (Fig. 5E left panel and 5G) and mock-infected samples (Fig. 5E right panel and 5G). In contrast, increasing the concentration of scrambled siRNA control (25nM and 45nM) did not affect the phosphorylation levels of p38 MAPK (Fig. 5E and 5G), suggesting a correlation between MINK expression levels and p38 MAPK phosphorylation levels. As such, these results confirmed that MINK plays a role in the downstream triggering of p38 MAPK phosphorylation during EV71 replication.

### The p38 MAPK signalling pathway is essential for EV71 replication in RD cells

**Fig 6:**
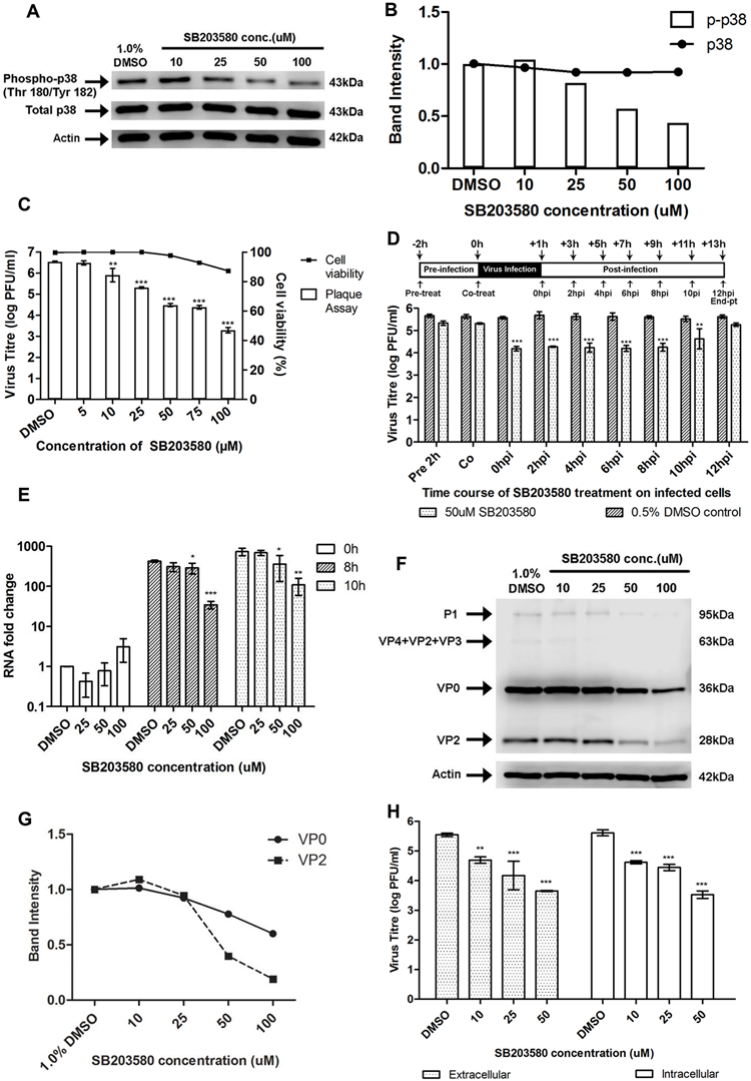
Treatment with p38 MAPK inhibitor (SB203580) inhibits EV71 replication at the viral protein synthesis stage. (A) Mock-infected RD cells were treated with SB203580 at different concentrations (10, 25, 50 and 100μM) or 1.0% DMSO (negative control) and cell lysates were harvested for Western blotting at 6h post-treatment. β-actin was included as a loading control. (B) Quantification of phospho-p38 MAPK (Thr180/Tyr182) and total p38 protein bands with reference to actin control bands (for each SB203580 concentration) and untreated control using ImageJ Gel Analysis program. (C) Cell viability of SB203580-treated cells and untreated control cells were measured using alamarBlue assay at 12h post-treatment. Values obtained were normalised against DMSO control. Virus titres in the supernatant of cells (denoted by bars) treated with varying concentrations of SB203580 post-adsorption were analysed via viral plaque assay. Error bars represent standard deviation (SD) of triplicate data. Statistical analyses were performed using one-way ANOVA and Dunnett’s test (Graphpad software) against untreated control **P < 0.01 (n = 3), *** *P* < 0.0001 (n = 3) versus 1.0% DMSO control. (D) RD cells were treated with 50uM SB203580 (p38 MAPK inhibitor) at different time points before and after infection in time-of-addition assay. Cell supernatants were harvested at 12hpi for quantification via viral plaque assays. Time-of-addition assay indicates that SB203580 acts between 2hpi and 10hpi to inhibit EV71 replication. In the co-treatment assay, SB203580 was added with the virus and no significant inhibition of EV71 infection was observed. Statistical analyses were performed using one-way ANOVA and Dunnett’s test (Graphpad software) against untreated control **P < 0.01 (n = 3), *** *P* < 0.0001 (n = 3) versus 0.5% DMSO control. (E) EV71 RNA synthesis was sensitive to SB203580 treatment. Quantitative RT-PCR assay revealed significant reduction in levels of EV71 RNA across increasing SB203580 concentration. Total RNA was extracted for all samples at 0, 8 and 10hpi and EV71 RNA levels were measured. C_T_ values were normalised against actin and relative quantification of viral RNA level was determined. The ΔΔCt data were calculated from three independent experiments and error bars represent standard deviation for triplicate data sets. Fold difference of viral RNA for all samples was calculated relative to the RNA level in the DMSO control at 0hpi. Statistical analyses were carried out using one-way ANOVA with Dunnett’s test (Graphpad software). *P <0.05, **P <0.01 and *** *P* < 0.0001 (n = 3) vs the respective 1.0% DMSO control at each time-point. (F) Viral protein expression levels upon SB203580 treatment. EV71-infected RD cells were treated with SB203580 and cell lysates were harvested for Western blotting at 8h post-treatment. VP0 and VP2 viral protein expression was observed to decrease with increasing concentration of the p38 MAPK inhibitor. (G) Band intensities of VP0 and VP2 upon SB203580 treatment. The band intensities representing VP0 and VP2 protein expression level were quantitated with reference to actin control bands (for each concentration) and DMSO control using ImageJ Gel Analysis program. (H) Extracellular and intracellular virion levels upon p38 MAPK inhibition. Extracellular EV71 virions in the supernatant and intracellular virus particles were harvested separately at 12hpi for viral plaque assay to assess the effect of SB203580 treatment on virus packaging and release. p38 MAPK inhibition resulted in significant reduction in both intracellular and extracellular virions. Statistical analysis was performed using one-way ANOVA with Dunnett’s test (Graphpad software). **P <0.01, *** *P* < 0.0001 (n = 3) versus 1.0% DMSO control.

To ascertain the role of p38 MAPK signalling pathway in EV71 replication, the p38 MAPK inhibitor SB203580 [30] was examined for its impact on EV71 replication. To verify the efficacy of SB203580 in inhibiting the p38 MAPK pathway, we measured the phosphorylation levels of p38 MAPK via Western blot analysis. A dose-dependent reduction in the phosphorylation of p38 MAPK was observed with increasing concentration of SB203580 and significant inhibition was noticed at a concentration of 50μM (Fig. 6A and 6B). Consistent expression levels of total p38 MAPK at different concentrations of SB203580 suggested that SB203580 inhibited p38 MAPK phosphorylation but not its expression.

Having confirmed the effectiveness of SB203580 in inhibiting the phosphorylation of p38 MAPK, RD cells were post-treated with SB203580 and the production of progeny virus in the culture supernatant was assessed via viral plaque assay. A significant dose-dependent reduction in the progeny virus production was observed after treatment with SB203580 with a 1.2-, 2.2- and 3.3 log reduction at 25 μM, 75 μM and 100 μM, respectively (Fig. 6C). In addition, cell viability of SB203580-treated cells was assessed to rule out the possibility of reduced viral growth due to cytotoxicity after treatment with SB203580. Our results from the alamarBlue cytotoxicity assay indicated that the concentration range of SB203580 used in this study did not lead to significant reductions in cell viability and hence, the dose-dependent reduction of EV71 virus titre by SB203580 was not complicated by its cytotoxic effects (Fig. 6C). Together, these results indicated that blockage of the p38 MAPK signalling pathway can significantly reduce viral growth and hence, p38 MAPK pathway is essential for EV71 propagation.

### Inhibition of the p38 MAPK signalling pathway affects viral protein synthesis

To further validate the involvement of p38 MAPK signalling pathway downstream of MINK in the synthesis of viral proteins, time-of-addition studies were conducted to identify the window period in the EV71 replication cycle when the p38 MAPK inhibitor, SB203580 exerts its antiviral effects. 50 μM of SB203580 was added at different time points before and after infection with EV71 (Fig. 6D). All cell culture supernatants were collected for viral plaque assay at 12h post-infection. From the results in Fig. 6D, pre-treatment of cells with the p38 MAPK inhibitor for 2h prior to EV71 infection showed minimal inhibitory effect against viral infection. A significant reduction in EV71 titres was observed when SB203580 was added at 6h post-infection. At 10h post-infection, the antiviral activity of SB203580 was reduced. This suggested that the blocking of p38 MAPK signalling pathway inhibits EV71 replication during the early phase after viral entry, between 0h and 8h post-infection. A co-treatment assay was conducted to complement data from the pre-treatment assay to determine if SB203580 affected viral binding and entry into the cells. Co-treatment of EV71 with SB203580 failed to inhibit viral infection, further confirming the involvement of p38 MAPK signalling pathway in the post-entry stage in EV71 replication cycle.

To reaffirm our hypothesis that MINK affects viral protein synthesis via p38 MAPK signalling pathway, the effect of p38 MAPK inhibition on viral RNA production, protein synthesis and viral release was investigated. In the analysis of viral RNA replication in SB203580-treated infected cells, fold change in RNA level for all samples was calculated relative to that in the DMSO control at 0h post-infection. Comparison of the relative RNA level was made between the samples treated the p38 MAPK inhibitor (SB203580) and the respective DMSO control at each time-point. Similar to what was observed for the siRNA knockdown of MINK (Fig. 3B), there was increase in viral RNA level between time-points and viral RNA level in SB203580-treated cells was significantly lower than that in the DMSO control at both 8h and 10h post-infection, with fold reductions of 12.4 and 9.0 at 8h and 10h post-infection at 100μM concentration, respectively (Fig. 6E). These results indicated that the blocking of p38 MAPK pathway also has inhibitory effects on the production of viral RNA. Significant reduction in the structural protein (VP0 and VP2, Fig. 6F and 6G) and non-structural protein levels (3D protein, S2D and S2E Fig) were also observed with increasing concentrations of SB203580. The extent of the reduction in 3D protein level upon SB203580 treatment corresponded to the decrease in the viral RNA levels. Hence, we have shown that p38 MAPK signalling pathway is involved in the viral protein synthesis stage.

As p38 MAPK signalling has been shown to play a critical role in the viral progeny release of coxsackievirus B3 (CVB3) [31], we were interested to know if p38 MAPK was also involved in the viral release of EV71 using the p38 MAPK inhibitor (SB203580). Intracellular and extracellular EV71 virions were quantified at 12h post-infection to determine whether p38 MAPK plays a role in viral packaging and release. Although a significant reduction of ~1.9 log was observed in the extracellular virions upon the inhibition of p38 MAPK at 50μM, a significant reduction in virus titre was also observed in the intracellular virions (Fig. 6H). Hence, we concluded that in accordance to what was observed with the siRNA knockdown of MINK, the reduction in the amount of virus released was not due to a blockage in the virus release process upon the inhibition of p38 MAPK signalling, but was due to a decrease in the total production of virus particles.

Together, these results coincided with our previous observations with the siRNA knockdown of MINK (Fig. 3) and supported our hypothesis that p38 MAPK signalling pathway is very likely to be involved downstream of MINK in regulating EV71 viral protein synthesis.

### MINK/p38 MAPK signalling positively regulates EV71 viral protein translation efficiency

**Fig 7:**
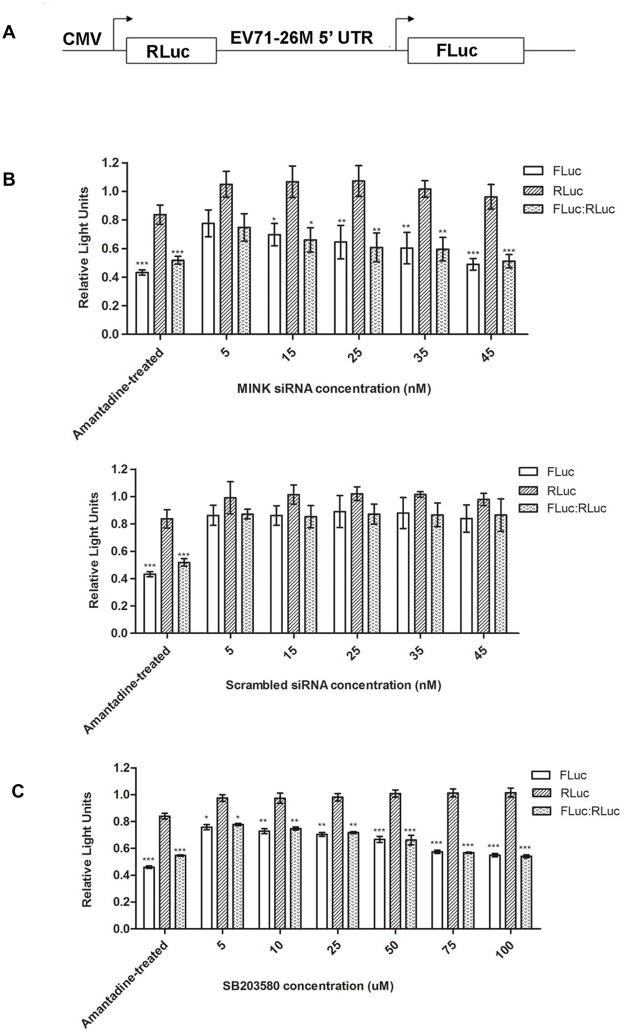
Silencing of MINK and inhibition of p38 MAPK phosphorylation reduces translation efficiency of EV71 IRES. (A) Schematic diagram of the bicistronic construct containing the *Renilla* luciferase (RLuc) and firefly luciferase (FLuc) genes controlled by the cytomegalovirus (CMV) promoter and 5’ UTR of EV71–26M [67], respectively. (B) Effect of knockdown of MINK on EV71 IRES activity. RD cells were pre-treated with MINK or scrambled siRNA. Three days after transfection, the bicistronic construct was then transfected into the cells. Luciferase activity was measured 24h after transfection. Amantadine, an inhibitor of EV71 IRES [33], was added to untreated cells to serve as negative control for IRES activity. Untreated cells that were transfected with the bicistronic construct were used as positive control. The FLuc/RLuc ratio for each sample were normalised to the FLuc/RLuc ratio of untreated control. Dose-dependent reduction in relative translation efficiency of the IRES was observed in MINK siRNA-treated cells. Error bars represent standard deviation of triplicate data sets. Statistical analyses were performed using one-way ANOVA with Dunnett’s test (Graphpad software). *P < 0.05, **P < 0.01 and *** *P* < 0.0001 versus untreated control. (C) Effect of p38 MAPK inhibition on EV71 IRES activity. Relative translation efficiency was determined as the ratio of FLuc to RLuc for each sample and the FLuc/ RLuc ratio for each sample were normalised to the FLuc/RLuc ratio of DMSO control, expressed as percentage. Error bars reflect the standard deviation of triplicate data sets. Transfected cells with the bicistronic construct without drug treatment (DMSO control) was used as positive control. Error bars represent standard deviation of triplicate data sets. Statistical analyses were performed using one-way ANOVA with Dunnett’s test (Graphpad software). *P < 0.05, **P < 0.01 and *** *P* < 0.0001 versus 1.0% DMSO control.

Translation initiation of the uncapped EV71 viral RNA is known to be mediated by a cap-independent mechanism which involves the IRES situated in the 5’ UTR of the viral genome [32]. Since the silencing of MINK reduced viral protein synthesis, we want to investigate if MINK/p38 MAPK signalling was involved in the regulation of IRES efficiency during viral protein synthesis. To determine if MINK/p38 MAPK is involved in IRES-mediated translation of EV71 transcripts, a bicistronic luciferase reporter construct containing the EV71 IRES and two luciferase reporter genes (firefly luciferase and *Renilla* luciferase, Fig. 7A) was transfected into cells. The expression of *Renilla* luciferase (RLuc) is dependent on the cap-dependent mechanism, while the translation of the firefly luciferase (FLuc) is IRES-dependent. The translation efficiency directed by the 5’ UTR of EV71 was determined by comparing the level of FLuc with the level of RLuc after the transfection of the non-replicating bicistronic luciferase reporter construct into RD cells that had been pre-treated with MINK siRNA. 0.5mg/ml of amantadine was added as a negative control (Amantadine-treated) to inhibit the EV71-IRES [33] while untreated cells serve as a positive control. MINK siRNA-treated cells showed a dose-dependent reduction in the EV71 IRES activity (FLuc level) and the relative translation efficiency of the IRES with significant decrease observed at 15nM (Fig. 7B). Cells treated with non-targeting siRNA (scrambled siRNA) showed minimal effect on the EV71 IRES activity as the relative translation efficiency remained relatively constant across the concentrations of scrambled siRNA with a non-significant decrease of 0.2 RLU at 45nM. In addition, a comparison of the RLuc levels of the scrambled control and MINK siRNA-treated cells indicated that the siRNA knockdown of MINK had minimal effect on cap-dependent translation.

To further confirm our hypothesis that p38 MAPK is involved in the translational regulation of viral transcripts downstream of MINK activation, RD cells were transfected with the bicistronic luciferase reporter construct before they were treated with different concentrations of SB203580. Results showed significant dose-dependent reduction in the relative translation efficiency of EV71 IRES in cells treated with SB203580 (Fig. 7C) relative to the DMSO control without affecting the cap-dependent translation. Thus, these data indicated that MINK/p38 MAPK is involved in the positive regulation of IRES-mediated translation of EV71 RNA.

### Silencing of MINK and p38 MAPK inhibition reduced hnRNP A1 signals in the cytoplasm upon EV71 infection

In a recent publication, phosphorylated eIF4E has been reported to improve viral protein translation via the IRES of rhinovirus by modulating the eIF4G:IRES interaction [34]. Hence, to investigate if eIF4E was the downstream effector of the MINK/p38 MAPK pathway that led to the increase in IRES-mediated protein translation efficiency, a time course study was conducted to examine the activation profile of downstream substrates of p38 MAPK in response to EV71 infection. Contrary to the findings in the rhinovirus IRES-mediated protein translation, we did not detect any increase in eIF4E phosphorylation levels upon EV71 infection. Instead, eIF4E protein expression and phosphorylation levels were observed to decrease upon EV71 infection (S4A and S4B Fig), suggesting that eIF4E might not be essential for EV71 replication.

**Fig 8:**
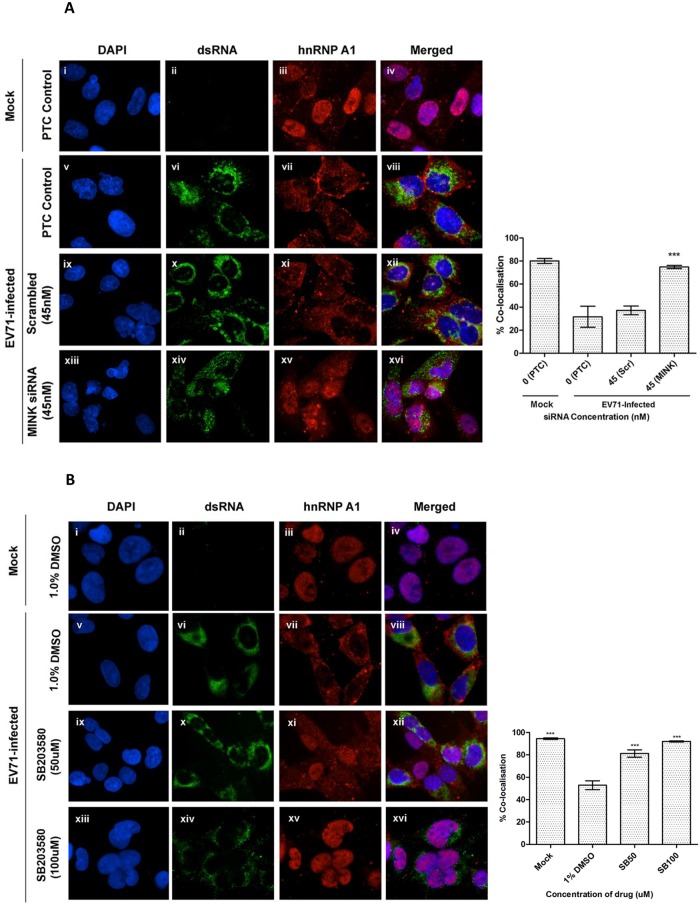
MINK silencing and p38 MAPK inhibition in EV71-infected cells inhibits cytoplasmic localisation of hnRNP A1. (A) RD cells were pre-treated with MINK targeting and scrambled siRNA and subjected to infection with EV71. siRNA-treated cells were fixed and the subcellular localisation of hnRNP A1 (red), an IRES-transacting factor, was investigated by indirect immunofluorescence assay. Immunofluorescence detection of double-stranded RNA (dsRNA, green) with the nuclei stained with DAPI (blue) was shown to indicate EV71 infection. The images were taken at 100X magnification. Colocalisation quantification was based on the Manders Overlap Coefficient (MOC) using whole-cell immunofluorescence (WCIF) ImageJ software [36] and represented as percent colocalisation at the respective siRNA concentrations. Error bars represent the standard deviation of duplicate data. (B) RD cells were subjected to infection with EV71 and post-treated with SB203580 (p38 MAPK inhibitor) for 8h. SB203580-treated cells were fixed and the subcellular localisation of hnRNP A1 (red) was investigated by indirect immunofluorescence assay. Mock-infected and DMSO-treated cells were included as infection and solvent control, respectively. The images were taken at 100X magnification. Colocalisation quantification was based on the MOC using WCIF ImageJ software and represented as percent colocalisation at the respective drug concentrations. Error bars represent the standard deviation of duplicate data.

Since eIF4E was unlikely to be the effector of the MINK/p38 MAPK pathway in our study, we hypothesized that the MINK/p38 MAPK signalling pathway might activate an IRES tran-sacting factor (ITAF) downstream, which thus resulted in the positive regulation of the EV71 IRES translation efficiency. Heterogenous nuclear ribonucleoprotein A1 (hnRNP A1) is predominantly a nuclear protein but shuttles back and forth between the nucleus and cytoplasm. It has been reported that hnRNP A1 act as an ITAF that relocalises in the cytoplasm where it interacts with the EV71 IRES upon infection, promoting its translation efficiency [20]. As previous study has also shown that p38 MAPK signalling is implicated in the cytoplasmic accumulation of hnRNP A1 in uninfected cells [35], we were interested to know whether MINK/p38 MAPK signalling modulate the EV71 IRES activity by altering the subcellular localisation of hnRNP A1. Fig. 8A shows the immunofluorescence staining of hnRNP A1 in EV71-infected cells at 8h post-infection. The degree of colocalisation between the hnRNP A1 protein (stained with rhodamine) and the cell nucleus (stained with DAPI) was quantified using Manders coefficient [36]. The level of the hnRNP A1 in the nucleus increased significantly upon the siRNA-knockdown of MINK (74.8% colocalisation, Fig. 8A xiii-xvi) compared to the scrambled control (37.2% colocalisation, Fig. 8A ix-xii), resembling the state of hnRNP A1 in mock-infected cells (80.1% colocalisation, Fig. 8A i-iv). These data demonstrated that the silencing of MINK reduced the hnRNP A1 signals in the cytoplasm as the degree of colocalisation between the hnRNP A1 signals and DAPI signals in the nucleus increased upon the siRNA knockdown of MINK.

Similarly, we investigated the subcellular localisation of hnRNP A1 upon the inhibition of p38 MAPK signalling pathway with the use of a p38 MAPK inhibitor (SB203580). Fig. 8B shows the immunofluorescence staining of hnRNP A1 in EV71-infected cells at 8h post-infection. The level of the hnRNP A1 in the nucleus increased significantly upon SB203580 treatment at 50μM (81.3% colocalisation, Fig. 8B ix-xii) compared to the DMSO control (52.9% colocalisation, Fig. 8B v-viii). In addition, the level of hnRNP A1 in the nucleus was even higher in cells treated with 100μM of SB203580 (92% colocalisation, Fig. 8B xiii-xvi), resembling the state of hnRNP A1 in mock-infected cells (94.5% colocalisation, Fig. 8B i-iv). These data demonstrated that the inhibition of p38 MAPK reduced the hnRNP A1 signals in the cytoplasm as the degree of colocalisation between the hnRNP A1 signals and DAPI signals in the nucleus increased upon SB203580 treatment. Together, we have shown in our study that it is likely that hnRNP A1 was one of the targets downstream of MINK/p38 MAPK signalling which is involved in promoting the translation efficiency of EV71 IRES as the relocalisation of hnRNP A1 to the cytoplasm where it binds to the IRES of EV71 RNA is required to facilitate translation initiation of viral RNA.

### Cytoplasmic localisation of hnRNP A1 resulted from MINK/p38 MAPK signalling was not stimulated by Mnk1 activity

As Mnk1 has been implicated in the cytoplasmic localisation of hnRNP A1 downstream of p38 MAPK signalling in uninfected cells [37], we were interested to know if Mnk1 was the p38 MAPK substrate that triggered the increase of hnRNP A1 signals in the cytoplasm during EV71 infection. A time course study was first conducted to examine the activation profile of Mnk1. Compared to the mock-infected cells, EV71 infection resulted in increased Mnk1 phosphorylation levels at 8h post-infection with relatively constant Mnk1 protein expression levels (S4A and S4B Fig). To determine if Mnk1 phosphorylation was triggered by the replication of the virus, RD cells were exposed to UV-inactivated EV71. Cells exposed to UV-inactivated EV71 showed constant phosphorylation level of Mnk1 throughout the 12h time course (S4A and S4B Fig). This indicated that attachment of the virions to cell surface receptors or virus entry process was not sufficient to trigger the phosphorylation of Mnk1, which corresponded to what was observed previously for the activation of MINK and p38 MAPK upon EV71 infection (Fig. 4 and 5).

To investigate if Mnk1 was involved in the mechanism elucidated in this study, we blocked Mnk1 kinase activity with a selective Mnk inhibitor (CGP57380) in RD cells and conducted Western blot analyses with phospho-eIF4E antibody to determine the effectivity of the drug. At 50μM, CGP57380 was effective in blocking the kinase activity of Mnk as indicated by the intensity of the phospho-eIF4E band (S4C and S4D Fig). However, there was no changes in the viral protein levels (VP0 and VP2, S4E and S4F Fig) as well as the virus titre (S4G Fig) at effective concentrations of 50μM and 100μM. Moreover, inhibition of Mnk1 did not alter the subcellular localisation of hnRNP A1 (S4H Fig). Thus, it is likely that the cytoplasmic localisation of hnRNP A1 as a result of MINK/p38 MAPK signalling was not triggered by Mnk. Further investigation is required to identify the p38 substrates involved downstream of MINK/p38 MAPK signalling that modulates the cytoplasmic localisation of hnRNP A1.

## Discussion

Viruses have been known to hijack cellular signalling pathways during viral infection to facilitate events such as viral entry, inhibition of apoptosis and to escape antiviral activities elicited by host factors such as interferon [38,39,40]. In relation to EV71, the recent work conducted by Hussain and colleagues (2011) identified several kinases with potential involvement in the clathrin-mediated endocytosis of EV71 into RD cells [26]. Thus, host kinases may represent a family of host factors which may play roles in facilitating EV71 replication and have the potential of becoming a viable antiviral target.

In this study, a novel mitogen-activated protein kinase kinase kinase kinase (MAP4K) has been identified from our primary screen for further investigation as interest in the MAPK family of kinases was sparked by previous studies on the involvement of MAPK family members in viral replication [41] and the pathogenesis of viruses [42,43]. Here, we demonstrated that MINK plays an essential role in EV71 replication and may be a potential target for antiviral development.

Infected cells have been shown to induce the activation of kinases in early events of viral infection, such as the activation of PAK1 upon Myxoma Virus infection [44]. Since MINK is a MAP4K, an upstream regulator in the MAPK signalling cascade, it was hypothesised that MINK might have been activated by early events of viral infection, such as the attachment of the virus or the uncoating of the virions. Activation profiles of MINK and entry assays have revealed that virus attachment and clathrin-mediated entry was not sufficient in triggering the activation of MINK. Instead, active replication of the virus and the accumulation of viral RNA or proteins might be potential inducers of MINK phosphorylation. To determine the exact cellular or viral factor that activated MINK upon infection, further studies such as a co-immunoprecipitation could be employed to identify viral factors that interact with MINK protein to trigger its activation.

Investigation into the stage of involvement of MINK in EV71 replication has also revealed interesting findings on the functional role of MINK in the propagation of EV71. Prior to our work, MINK has been suggested to play a role in the reorganisation of cytoskeleton such as actin filaments [22] which have been implicated in the formation of clathrin-coated vesicles in endocytosis pathways [45,46]. Since EV71 is known to enter RD cells via clathrin-mediated endocytosis [26], we first hypothesised that actin rearrangement induced by the activation of MINK may play a role during the clathrin-mediated entry of EV71. However, results from the viral entry study via viral RNA transfection were contrary to this hypothesis as silencing of MINK continued to cause inhibition of progeny virus production despite bypassing the normal entry pathway. Results from the real time RT-PCR analysis have also indicated that the silencing of MINK has an effect on the viral RNA levels. During EV71 replication, protein translation of the viral genomic RNA has to precede viral RNA replication as the production of non-structural proteins such as the RNA-dependent RNA polymerase (Protein 3D) is essential for the synthesis of viral RNA. As such, synthesis of viral proteins was also investigated upon the silencing of MINK, structural viral protein (VP0 and VP2) and non-structural protein (3D) levels was significantly inhibited in a dose-dependent manner with increasing MINK siRNA concentrations. However, since VP0 and VP2 are both products of proteolytic cleavage of the viral polyprotein, there is a possibility that the siRNA knockdown of MINK affected the proteolytic cleavage efficiency which led to the reduction in VP0 and VP2 levels. The VP2 antibody (MAB979) used in this study recognizes a specific epitope on the VP2 protein [47], hence, larger incomplete processed viral polyproteins containing the VP2 epitope can also be detected. In this study, we have demonstrated that the protein levels of larger incomplete processed viral polyproteins (sizes coincided to that of P1 and VP4+VP3+VP2) [48] also showed similar trend to that of VP0 and VP2 protein levels where a dose-dependent reduction in the incomplete processed viral polyproteins levels was observed with the siRNA knockdown of MINK but not in the scrambled control. This narrowed down the potential target event of MINK involvement in the process of viral protein synthesis and not the proteolytic cleavage of the viral polyprotein. In addition, siRNA knockdown of MINK did not lead to an accumulation of intracellular virus, but instead, resulted in a global decrease in both extracellular and intracellular virus titres. This demonstrated that the silencing of MINK suppressed the production of progeny virus and not virus release.

Although the functional relationship between MINK and p38 MAPK in normal cellular processes [23, 49] has already been established, this association has not been reported in virus replication. Here, activation profile of p38 MAPK upon EV71 infection has suggested its essential role in EV71 replication and siRNA-mediated gene knockdown has demonstrated the correlation between MINK protein expression (consequently its activation) and p38 MAPK activation in the context of virus replication. The activation profile of MINK and p38 MAPK has also ascertained that p38 MAPK was activated downstream of MINK, which corresponded to what was reported in uninfected cellular conditions [23,49].

Involvement of p38 MAPK signalling pathway in virus replication has been well established and this signalling cascade has been reported to promote viral RNA synthesis and protein synthesis in some viruses such as the encephalomyocarditis virus (EMCV) [25], mouse hepatitis virus (MHV)[50] and hepatitis B virus [51]. As shown previously by Hirasawa and his colleagues (2003), p38 MAPK signalling pathway promotes viral protein synthesis but not viral RNA synthesis in EMCV, which also belongs to the same family as EV71. Their study has demonstrated that p38 MAPK signalling pathway facilitates EMCV protein synthesis by promoting the translation efficiency of the IRES in EMCV. Supporting this hypothesis, our study has demonstrated the crucial role of p38 MAPK in the propagation of EV71 and that the activation of the MINK/p38 MAPK signalling pathway promotes the translation efficiency of EV71 IRES during EV71 replication. Time-of-addition assay conducted in our study verified that p38 MAPK inhibitor, SB203580, was effective in inhibiting the early events of EV71 replication cycle post-infection and not late events such as virus release. Inhibition of p38 MAPK signalling also demonstrated an inhibition in the synthesis of EV71 viral proteins (3D, VP0 and VP2) and viral RNA replication which were in agreement with the results observed upon the silencing of MINK. Contrary to what was observed with coxsackievirus B3 (CVB3) [31], the inhibition of p38 MAPK did not block the release of the virus. Hence, this further supported the relationship between MINK and p38 MAPK signalling and the involvement of MINK/p38 MAPK in EV71 protein synthesis which resulted in a global decrease in the progeny virus production.

As mentioned in this study, EV71 has a type 1 IRES that requires eukaryotic initiation factors (eIFs) and IRES-specific transacting factors (ITAFs) to initiate viral protein translation [52]. Previous study on a poliovirus/rhinovirus chimera (PSRIPO) [34] has shown that signal transduction to Mnk1, a downstream substrate of p38 MAPK can favour viral, cap-independent translation via eIF4E phosphorylation and expression. Contrary to this finding, our data showed a down-regulation in the protein expression of eIF4E which led to a consequent reduction in its phosphorylation levels, suggesting that eIF4E phosphorylation and expression may not be crucial for EV71 replication. This finding was supported by another study [53] that has demonstrated that induction of miRNA-141 during EV71 infection down-regulated eIF4E in an attempt to suppress cap-dependent translation and promote the switch to cap-independent translation.

Apart from factor eIF4E, other canonical translation factor such as eIF2α has also been reported to play a role in Picornavirus replication [54]. Factor eIF2α is a 36kDa protein that contains a serine residue (Ser-51) which can be phosphorylated under nutrient deprivation or cellular stresses such as virus infection or heat-shock. GCN2, PKR, PERK and HRI have been shown to phosphorylated eIF2α in response to amino acid starvation, double-stranded RNA, protein misfolding at the endoplasmic reticulum and the absence of HEME, respectively [55]. Phosphorylation of eIF2α inhibits the GDP-GTP recycling catalysed by eIF2B, hindering the generation of the ternary complex Met-tRNAi-eiF2-GTP and binding of this complex to the 40S ribosome to initiate translation [56]. Although eIF2α is required for both cap-dependent and IRES-mediated protein translation, studies have shown that some viral IRES elements can translate independent of phosphorylation of eIF2α [57,58]. Consistent with studies on enteroviruses [32], we observed an increasing level of phosphorylated eIF2α at late times post-infection (S3A and S3B Fig). Published studies on poliovirus have also demonstrated that resistance to eIF2α phosphorylation increases as enteroviral infection progresses due to the cleavage of initiation factor eIF5B by the viral 3C protease. As such, the induction of eIF2α phosphorylation at the late time-points of EV71 infection may also serve to promote the viral protein synthesis indirectly by suppressing cellular cap-dependent protein synthesis [59]. Although p38 MAPK signalling has not been implicated in the phosphorylation of eIF2α and significant reduction in cap-dependent protein translation was not observed in our luciferase data, we have conducted brief experiments to investigate the relationship between MINK expression and the phosphorylation of eIF2α (S3C and S3D Fig). From the suppression of eIF2α phosphorylation upon the silencing of MINK, it is tempting to speculate that the phosphorylation of eIF2α may be a minor side effect of the activation of MINK that serve to promote EV71 protein synthesis. Future downstream studies have to be performed to elucidate the role and involvement of eIF2α in EV71 replication in relation to MINK. Nonetheless, our findings on these canonical translation initiation factors suggested that the increased EV71 IRES translation efficiency observed in our study might have resulted from the activation of ITAFs downstream of MINK/p38 MAPK signalling instead of the phosphorylation status of the eIFs.

**Fig 9:**
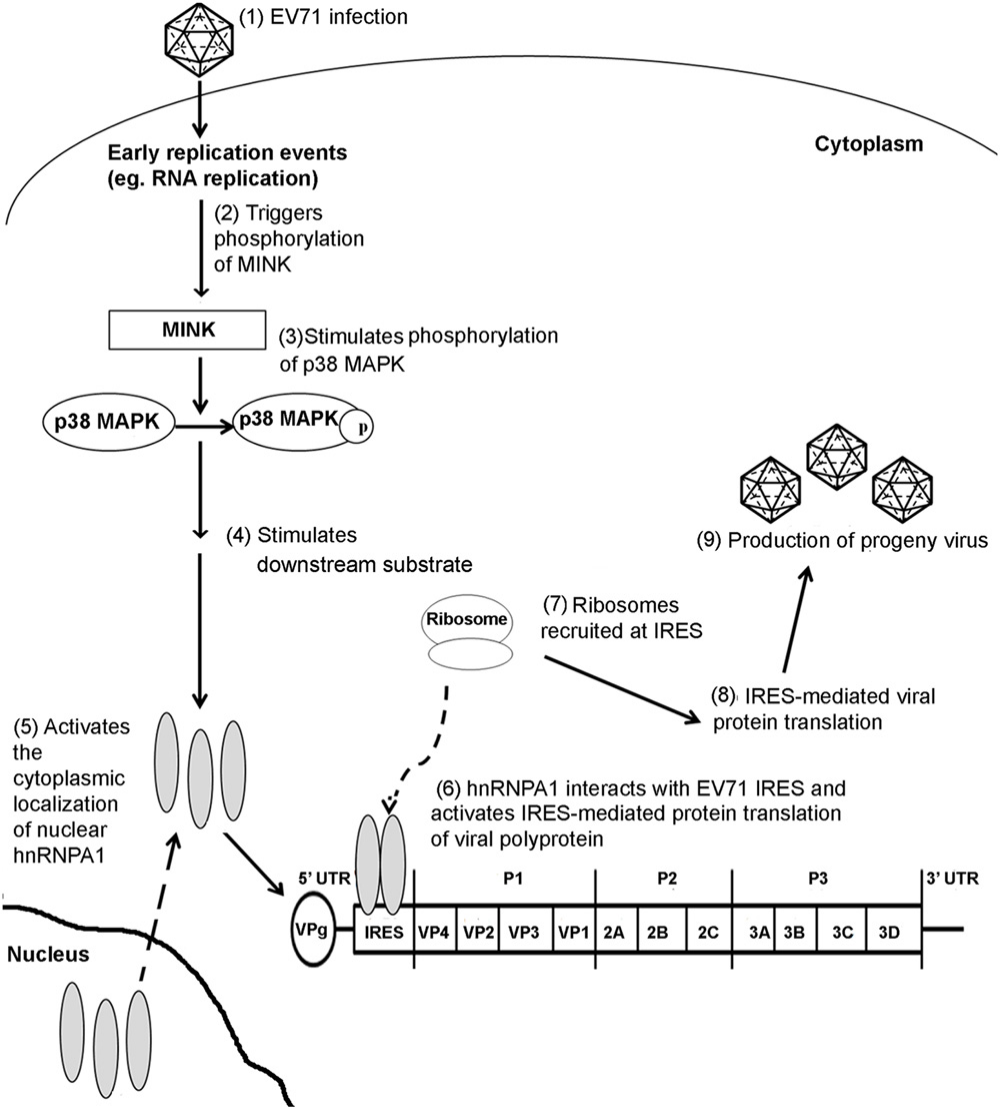
Proposed mechanism of action of MINK in the EV71 replication cycle. EV71 infection stimulates MINK activation which in turn triggers the phosphorylation of p38 MAPK downstream. The phosphorylation of p38 MAPK triggers a kinase cascade which results in the cytoplasmic relocalisation of hnRNP A1. hnRNP A1 binds to the viral IRES and promotes the recruitment of ribosomes at the IRES at the 5’ untranslated region (UTR) of EV71 genome, stimulating the IRES-mediated viral protein translation.

Members of the heterogeneous nuclear ribonucleoprotein (hnRNP) classes have been identified as trans-acting factors that control translation initiation of various cellular and viral mRNAs at the IRES [60]. Among the hnRNP family, hnRNP A1 has been reported to modulate the IRES-mediated viral protein translation of various viruses such as the human rhinovirus (HRV) [61] and EV71 [20]. Although, hnRNP A1 localises predominantly in the nucleus, it is able to shuttle between the nucleus and cytoplasm in a regulated manner [62]. Infection of cells with HRV and EV71 has shown to result in the cytoplasmic relocalisation of hnRNP A1 where it interacts directly with the viral IRES sequences [20]. Apart from picornaviruses, cytoplasmic accumulation of hnRNP A1 has also been reported to play a role in the positive regulation of human immunodeficiency virus (HIV) [63] and Sindbis virus (SINV) [20] viral RNA translation. In uninfected cells, activation of the p38 MAPK pathway upon osmotic shock or UV irradiation has been revealed to result in a phosphorylation-dependent cytoplasmic accumulation of hnRNP A1 [35]. Furthermore, a separate study has also demonstrated that the p38 MAPK interacts and regulates the subcellular localisation of hnRNP A1 in a Mnk1-dependent manner in senescent cells [37]. The cytoplasmic relocalisation of hnRNP A1 after EV71 infection may therefore also be dependent on the p38 MAPK pathway and its downstream substrate Mnk1 as in uninfected cells. In our study, subcellular localisation studies unravelled the relationship between MINK protein expression and hnRNP A1 localisation in the cells. Interestingly, we have found that the silencing of MINK upon EV71 infection did not result in the cytoplasmic accummulation of hnRNP A1 which was usually observed in infected cells. The nuclear retention of hnRNP A1 could be due to either lower levels of EV71 replication as a result of MINK silencing or a block in nuclear export signal [64] brought about by the siRNA knockdown of MINK. Similarly, the inhibition of p38 MAPK with a specific p38 MAPK inhibitor (SB203580) also resulted in the accumulation of hnRNP A1 signals in the nucleus. Although we have demonstrated that inhibition of the MINK/p38 MAPK signalling pathway reduced the hnRNP A1 signals in the cytoplasm that was observed in control EV71-infected cells, we have no direct evidence suggesting that MINK plays a direct role on the cytoplasmic relocalisation of hnRNP A1 where it binds directly to the IRES sequences of the viral genome to promote the IRES-mediated translation of the EV71 viral RNA. In addition, we have also shown that despite its activation during EV71, p38 MAPK substrate Mnk1 was not involved in the regulation of EV71 protein synthesis and the cytoplasmic relocalisation of hnRNP A1. Hence, the exact mechanism of how MINK/p38 MAPK signalling pathway affected the cytoplasmic relocalisation of hnRNP A1 during EV71 infection and the p38 MAPK substrates involved needs to be further established. Nonetheless, we have shown in this study that a novel host kinase (MINK) mediates the cap-independent translation of EV71 RNA, possibly by modulating the subcellular localisation of hnRNP A1, which further supports its propagation (Fig. 9). As such, MINK can be further explored as potential antiviral target for the inhibition of EV71 viral replication at the viral protein translation stage.

## Materials and Methods

### Cell line and viruses

Human rhabdomyosarcoma (RD) cells (CCL136TM, ATCC) cells were maintained in Dulbecco’s Modified Eagle’s Medium (DMEM, Sigma-Aldrich) enriched with 10% fetal calf serum (FCS, PAA Laboratories) in T75 at 37°C in an atmosphere of 5% CO_2_. Human Enterovirus 71 (EV71) strain H (VR-1432) was obtained from ATCC (GenBank accession no. AY053402.1) and EV71 strain 5865/sin/000009 (designated as strain 41, GenBank accession no. AF316321) was a kind gift from Dr Vincent Chow [65], Department of Microbiology, National University of Singapore. Coxsackivirus A6 (CA6, GenBank accession no. KC866983) and Echovirus 7 Wallace strain (Eo7-Wallace, GenBank accession no. AF465516) were obtained from the department collection and the viruses were propagated in RD cells. UV-inactivated EV71 was prepared by subjecting the virus stock to UV light irradiation for 2h before performing viral plaque assay to ensure complete inactivation.

### siRNA

Dharmacon siGENOME Human SMARTpool custom siRNA library targeting human serine/threonine kinases was obtained from Dharmacon RNA Technologies (Thermo Scientific, Dharmacon RTF # H-004405). The library contains a total of 47 siRNA cocktails with each cocktail consisting of 4 siRNA sequences targeting a specific gene and resuspended at a concentration of 2μM in 96-well plates. The separate and individual siRNA pools that were used to validate the hits identified from the screen was also obtained from Dharmacon (siGENOME SMARTpool): MINK (M-004861–03–0005), MAP4K2 (M-003587–01–0005). The sequences of the respective scrambled siRNA obtained from Origene are: 5’-GAUUAAACGCAUGGCCUUU-3’, 5’-GUAAGAGCACAAGUCGUGG-3’, 5’-UCUAGAAGACUUUGGAAGA-3’ and 5’-GGCUAUAUUCUCUGUUGAC-3’. For the deconvolution of MINK siRNA SMARTpool, individual siRNAs were also obtained from Dharmacon (MQ-004861–03–0005).

### siRNA reverse transfection

A reverse transfection protocol was used to perform the siRNA screen. The primary screen was performed in a 384-well format at a final concentration of 25nM siRNA using lμl of DharmaFECT-1 transfection reagent (Thermo Scientific) per well. Specific targeting siRNAs and scrambled siRNAs were dissolved in diethyl pyrocarbonate (DEPC)-treated reverse osmosis (RO) water to a stock concentration of 100μM. The siRNAs were then diluted to desired working concentrations with DharmaFect Cell Culture Reagent (DCCR) and DharmaFect-1 transfection reagent. The siRNAs were transfected into RD cells for 72h at 37°C in a 5% CO_2_ atmosphere prior to infection.

### Immunofluorescence assay

In the primary screen, cell monolayers were washed twice with PBS and fixed in cold absolute methanol (Sinopharm Chemical) at -20°C for 10 min at 12h post-infection. The cells were then washed thrice with PBS using the automated plate washer (Molecular Device) and incubated for 1h with 10μl of mouse monoclonal anti-EV71 antibody (#MAB979, Millipore). Cells were then washed thrice with PBS and incubated for another 1h with 10μl of secondary antibody, goat anti-mouse (Millipore) fluorescein isothiocyanate (FITC). DAPI (4’, 6-diamidino-2-phenylindole, Invitrogen) was used to stain cell nuclei for 15 min at room temperature. The images for the immunofluorescence assay were obtained using an automated Cellomics ArrayScan V^TI^ HCS System and the auto-focusing parameters were preset from Cellomics Arrayscan Instrument using module ‘Target Activation Bio-Application Version 3’. For the validation of primary screening hits, stained cells were visualised and images were captured using an Olympus 1X81 inverted fluorescence microscope (Olympus).

For the study on hnRNP A1 localisation, RD cells grown on coverslips were pre-treated with either scrambled or MINK siRNA for 72h. At 72h post-transfection, the cells were infected at MOI 1 for 8h. After washing thrice with PBS, the mock-infected and virus-infected cells on the coverslip were fixed with 4% paraformaldehyde (Sigma-Aldrich) at room temperature for 15 min. After three washes with PBS, the cells on the coverslip were permeabilised in 0.1% Triton X-100 at room temperature for 10 min and washed for another three times with PBS. The samples were then blocked in PBS containing 5% bovine serum albumin (BSA, MP Biomedicals) for 1h at 4°C and then incubated with primary antibodies at appropriate dilution: rabbit monoclonal anti-hnRNP A1 antibody (#ab177152, Abcam) and mouse monoclonal anti-dsRNA antibody (SCICONS) at 37°C for 1h. Upon removal of the primary antibodies, the samples were washed thrice with PBS. The samples were then incubated with secondary antibodies at appropriate dilution: Rhodamine-conjugated goat anti-rabbit IgG (Millipore), fluorescein isothiocyanate (FITC)-conjugated goat anti-mouse IgG (Millipore) at 37°C for 1h. Subsequently, Duolink In Situ mounting medium with DAPI (OLink BioSciences) was used to stain the cell nuclei and mount the coverslip on a microscope slide. The specimens were viewed with a 100X oil immersion lens with a numerical aperture (NA) of 1.6 of Olympus IX81. Colocalisation was quantified based on fluorescence microscopy images using the NIH ImageJ software (Wright Cell Imaging Facility) via the colocalisation analysis plug-in. Manders overlap coefficient (MOC) represents the proportion of normalised pixels in which the two signals overlap and was used as a measure of colocalisation [36]. MOC ranges from 0 for no colocalisation between the signals and 1 for perfect overlap. The percentage of colocalisation between the hnRNP A1 signals and nucleus was determined based on the MOC.

### Viral plaque assays

Plaque assay was performed on monolayers of RD cells in 24-well plates for the quantification of virus titre. Supernatants from EV71-infected samples were diluted in 10-fold dilutions with DMEM supplemented with 2% FCS before infection. The cells were incubated with the supernatant for 1h at 37°C with gentle rocking during the adsorption period. Infected cells were washed twice with PBS and overlaid with 1% carboxymethyl-cellulose (CMC) in DMEM with 2% FCS. Plaques were allowed to form for 4 days at 37°C in an atmosphere of 5% CO_2_. After which, the cells were fixed and stained with 10% paraformaldehyde/1% crystal violet (Sigma-Aldrich) solution. Virus titres were expressed as plaque forming units per millilitre (PFU/ml).

### Cell extract preparation and western blot analyses

Whole cell extracts were prepared with ice cold lysis buffer cocktail containing Halt phosphatase and protease inhibitor cocktail (100X), 0.5M EDTA (100X) and Mammalian Protein Extraction Reagent (mPER) (Thermo Scientific). For samples separated using the Phos-tag technology (Phos-tag AAL-107, Wako), cells were lysed in mPER with the addition of complete protease inhibitor cocktail tablet (Roche). Extracts were separated on classical SDS-PAGE or using the Phos-tag technology (Wako, Phos-tag AAL-107) which allows the mobility shift detection of phosphorylated proteins in SDS-PAGE.

The proteins in the cell lysates were resolved by SDS-PAGE and immobilised on nitrocellulose membrane (Bio-Rad). Blocking was performed at room temperature in 5% BSA for 1h and then incubated with one of the following primary antibodies overnight at 4°C: Rabbit polyclonal anti-MINK (#ab86385), rabbit polyclonal anti-MAP4K2 (#ab82870), rabbit polyclonal anti-phosphoeIF4E (S209, #ab76256), mouse monoclonal anti-eIF4E (#ab130210), rabbit polyclonal anti-phospho-eIF2α (S51, #ab47769) and anti-eIF2α (#ab5269) were obtained from Abcam. Mouse monoclonal anti-EV71 (#MAB979) was obtained from Millipore. Rabbit polyclonal anti-p38 (#9212), rabbit polyclonal anti-phospho-p38 (p-p38, Thr180/Tyr182, #9211), rabbit monoclonal anti-MNK1 and rabbit polyclonal anti-phospho-Mnk1 (p-Mnk1, Thr 197/202, #2111) antibodies were obtained from Cell Signalling Technology. Mouse monoclonal anti-3D antibody (#GTX630193) was obtained from Genetex. The blots were subsequently incubated with the following secondary antibodies at 37°C for 1h: Polyclonal Goat anti-mouse IgG (H+L) Horseradish peroxidase (HRP, Thermo Scientific) and polyclonal Goat anti-rabbit IgG (H+L) HRP (Thermo Scientific). SuperSignal West Dura chemiluminescent substrate (Thermo Scientific) was used in the enhanced chemiluminescent detection (ECL) of the protein bands on the membranes. Restore PLUS stripping buffer (Thermo Scientific) was used for effective removal of bound antibodies on the blot for reprobing. Chemiluminescent Western blot imaging is done using C-digit Chemiluminescence Western Blot Scanner (LI-COR).

### Cell viability assay

Cell viability profiles of siRNA-treated or drug-treated cells were assessed using the AlamarBlue reagent (Invitrogen) as recommended by the manufacturer’s protocol. AlamarBlue reagent was added to each well and the plates were incubated at 37°C supplemented with 5% CO_2_ for 4h and fluorescence measurements were taken using the Infinite 200 series microplate reader (Tecan). The measurements were performed at excitation wavelength of 570 nm and emission wavelength of 585 nm.

### Infection inhibition assay

Monolayers of RD cells were treated with increasing concentrations of antibody to SCARB2 (5, 25 or 50μg/ml, #AF1966, R&D Systems) for 30min at 37°C. As a control, a normal goat IgG (#AB-108-C, R&D Systems) was used in a similar assay. The cells were washed with cold PBS thrice and infected with EV71 at MOI 1 at 4°C for 1h. After 1h of incubation, the cells were washed with cold PBS thrice and incubated at 37°C supplemented with 5% CO_2_ for 6h before the cells were lysed.

### Viral RNA transfection into RD cells

Viral RNA was isolated and purified from EV71 viral supernatants using the QIAamp viral RNA minikit (Qiagen) according to the manufacturer's instructions. siRNA-treated RD cells were transfected with 2μg of EV71 viral RNA. During the transfection, EV71 viral RNA was diluted in reduced serum OPTI-MEM I (Gibco, Invitrogen) with the addition of Plus reagent (Invitrogen) added and left to incubate at room temperature for 5 mins. The diluted viral RNA solution was then added to the diluted Lipofectamine LTX and incubated at room temperature for 30 min. Growth medium was removed from the wells on the 24-well plate and 1ml of DMEM supplemented with 2% FCS was added into each well. Following incubation, viral RNA-Lipofectamine mixture was added into each well, giving a final amount of 2μg of viral RNA per well. Supernatants and cell lysates were harvested at indicated time-points post-transfection.

### Quantitative reverse transcription-PCR (qRT-PCR)

Total cell lysate was harvested at 0h, 8h and 10h post-infection and extraction was carried out using Total RNA Mini Kit (Blood/ Cultured Cell) (Qiagen). Extracted RNA was then subjected to Reverse-Transcription Real-Time Polymerase Chain Reaction (qRT-PCR).

Samples were assayed in a 25μl reaction mixture containing 12.5μl of SYBR Green (ThermoScientific) and reactions were carried out in the StepOne Plus Real-time PCR system (Applied Biosystems). The primer sequences for the 5’untranslated region (UTR) used in this study were: MD90 (5’-ATTGTCACCATAAGCAGCCA-3’) and MD91 (5’-CCTCCGGCCCCTGAATGCGGCTAAT-3’) which have been mentioned previously in [66].

### Treatment of cells with p38 MAPK inhibitor (SB203580) and Mnk1 inhibitor (CGP57380)

A working concentration of 10mM SB203580 (#5633S, Cell Signalling Technology) and CGP57380 (ab120365, Abcam) was prepared in dimethyl sulfoxide (DMSO). RD cells were infected with EV71 at MOI 1 for 1h before drug treatment for another 12h. After 12h of incubation, the supernatant were harvested for plaque assay.

### Time-of-addition studies

Time-of-addition assay was performed for SB203580 (#5633S, Cell Signalling Technology) on EV71-infected RD cells in 96-well plates. Cells treated with 0.5% DMSO were used as control. For the pre-treatment assay, cell monolayers were treated with 50μM of SB203580 for 2h at 37°C before being washed twice with PBS and infected with EV71 at MOI 1. After the 1h virus adsorption period, infected cells were washed with PBS and incubated in DMEM supplemented with 2% FCS at 37°C with 5% CO_2_ for 12h before supernatants were harvested for viral plaque assay.

For the co-treatment assay, SB203580 was added together with EV71 at MOI 1 to obtain a final SB203580 concentration of 50μM. After incubating the cells with this mixture for 1h, infected cells were washed with PBS and incubated in DMEM supplemented with 2% FCS at 37°C with 5% CO_2_ for 12h before supernatants were harvested for viral plaque assay.

For the post-treatment assays, RD cells were infected with EV71 at MOI 1 for 1h and 50μM of SB203580 was added every 2h post-infection (0h, 2h, 4h, 6h, 8h, 10h and 12h)

### Transfection of bicistronic construct of EV71-IRES

The bicistronic construct of EV71 IRES (Fig. 7A) was a kind gift from Professor Peter C McMinn, University of Sydney. The construct contains the 5’ untranslated region (UTR) of the EV71–26M strain and two reporter genes, *Renilla* luciferase (RLuc) and firefly luciferase (FLuc). The RLuc-reporter gene was positioned upstream of the EV71 5’ UTR controlled by the cytomegalovirus promoter (CMV). The firefly luciferase (FLuc) reporter gene is ligated downstream of the 5’ UTR which controls its expression [67].

1μg of plasmid DNA was introduced into RD cells using 2μL of Lipofectamine 2000 (Invitrogen) in Opti-MEM I Reduced serum (Gibco) according to manufacturer’s instructions. The cells were incubated for 12h and the media was replaced with DMEM 2% FCS growth medium. As a negative control, 0.5mg/ml of amantadine (Sigma-aldrich) was added to the media of untreated cells to inhibit the EV71 IRES activity. The cells were incubated at 37°C for another 12h before harvesting.

### Luciferase assay

Cells were washed twice with PBS and lysed with 100μL of Passive Lysis Buffer (Promega) for 15 min at room temperature with shaking. After complete lysis, the lysate was transferred to an opaque white 96-well plate. Luciferase activity was quantified using GloMax-Multi Detection System (Promega) according to manufacturer’s instructions.

### Statistical analysis

The Z’ factor, a statistical measurement of the distance between the standard deviations for the signal versus the noise of an assay, was employed as an indicator for the robustness of the screen. Experiments to determine the Z’ factor was conducted in a 384-well plate using positive controls where virus-infected cells were not treated with siRNA (growth media, transfection reagent and DCCR) and mock-infected cells as negative controls. The Z’ factor was then computed using the equation: 1-(3 x S.D. positive control + 3 x S.D. negative control) / (mean positive control—mean negative control). In other studies, one-way ANOVA test was used to compare the data and the results were considered to be significant if p ≤ 0.05.

### Accession numbers

Accession numbers for genes discussed in this study based on GenBank: PAK1 (NM_002576), MINK(NM_015716), MAP4K2(NM_004579), NEK3(NM_152720), NEK11(NM_145910), STK3(NM_006281), MAP2K5(NM_002757), NEK7(NM_133494),PAK2(NM_002577), MAP2K4(NM_003010), MAP4K3(NM_003618), MAP3K8(NM_005204),NEK9(NM_033116),MAP3K14(NM_003954),NEK1(NM_012224), STK10(NM_005990),PAK3(NM_002578),ALS2CR2(NM_018571),PAK4(NM_001014834),PAK7(NM_020341),PAK6(NM_020168),MAP3K2(NM_006609), MAP3K3(NM_002401), NEK6(NM_014397),MAP4K5(NM_006575),KIAA1361(NM_020791),MAP4K4(NM_004834),MAP2K3(NM_002756),NEK2(NM_002497),MYO3A(NM_017433), RP6213H19.1(NM_001042452),MAP3K5(NM_005923),OSR1(NM_005109),STK25(NM_006374),STK24(NM_003576),TNIK(NM_015028),CDC7(NM_003503), NEK4(NM_003157),FLJ23074(NM_001018046),MAP2K7(NM_145185),LYK5(NM_001003786),MAP3K4(NM_006724),JIK(NM_016281),MAP2K1(NM_002755),MAP2K6(NM_002758), MAP2K2(NM_030662), SLK(NM_014720)

Accession numbers for proteins discussed in this study based on UniProt: p38α(Q16539), p38β (Q15759), p38γ (P53778), EV71 VP0(A8Y8D7), VP2(Q9IWX5), VP1(Q80K68), Mnk1 (Q9BUB5), eIF4E (P06730), eIF2α (Q9BY44)

## Supporting Information

S1 Table List of targeted genes in the human serine/threonine kinase siRNA library used in primary screen. (PPTX)

S1 Fig Activation profile of MINK. (A) A sharp increase in MINK phosphorylation level was observed at 8h after the addition of virus in EV71-infected samples. (B) Low constant levels of phospho-MINK observed in mock-infected cells across the 8h time course. (C) Exposure to UV-inactivated EV71 showed a similar trend of phospho-MINK levels in mock-infected samples. (D) Quantification of phospho-MINK protein bands with reference to actin control bands (for each time-point) and 0h using ImageJ Gel Analysis program.
(PPTX)

S2 Fig Treatment with MINK siRNA and SB203580 reduces EV71 3D protein expression level. (A) Viral 3D protein expression levels upon the silencing of MINK. Viral protein expression was observed to decrease with increasing concentration of siRNA targeting MINK. (B) Band intensities of 3D protein upon siRNA knockdown of MINK. The band intensities representing 3D protein expression level were quantitated with reference to actin control bands (for each siRNA concentration) and 0nM using ImageJ Gel Analysis program. (C) Band intensities of 3CD protein upon siRNA knockdown of MINK. The band intensities representing 3CD protein expression level were quantitated with reference to actin control bands (for each siRNA concentration) and 0nM using ImageJ Gel Analysis program. (D) EV71 3D protein expression levels upon SB203580 treatment. EV71-infected RD cells were treated with SB203580 and cell lysates were harvested for Western blotting at 8h post-treatment. 3CD and 3D viral protein expression was observed to decrease with increasing concentration of the p38 MAPK inhibitor. (E) Band intensities of 3D and 3CD upon SB203580 treatment. The band intensities representing 3D and 3CD expression level were quantitated with reference to actin control bands (for each concentration) and 1.0% DMSO control using ImageJ Gel Analysis program.
(TIF)

S3 Fig Phosphorylation profile of eIF2α. (A) A sharp increase in eIF2α phosphorylation level was observed from 8h onwards after the addition of virus in EV71-infected samples. Low constant levels of phospho-eIF2α observed in mock-infected cells with slight increase at 12h. Exposure to UV-inactivated EV71 showed low basal phospho-eIF2α level across the 12h time course. (B) Quantification of phospho-eIF2α and total eIF2α protein bands with reference to actin control bands (for each time-point) and 0hpi using ImageJ Gel Analysis program. (C) Western blot analysis of the phosphorylation levels of eIF2α at 8hpi in infected cells pre-treated with either scrambled or MINK siRNA. β-actin was included as a loading control. (D) Quantification of phospho-eIF2α protein bands with reference to actin control bands (for each concentration) and PTC using ImageJ Gel Analysis program.
(PPTX)

S4 Fig Cytoplasmic localisation of hnRNP A1 resulted from MINK/p38 MAPK signalling was not stimulated by Mnk1 activity. (A) Western blot analyses of the activation profile of Mnk1 and eIF4E in cells subjected to the three treatments (EV71 infection, mock infection and UV-inactivated EV71). β-actin was included as a loading control. EV71 infection induced the phosphorylation of Mnk1 but downregulated eIF4E protein expression. (B) Quantification of phospho-Mnk1 (Thr197/202) protein bands with reference to actin control bands (for each time-point) and 0hpi using ImageJ Gel Analysis program. (C) Mock-infected RD cells were treated with CGP57380 at different concentrations (25, 50 and 100μM) or 1.0% DMSO (negative control) and cell lysates were harvested for Western blotting at 8h post-treatment. β-actin was included as a loading control. (D) Quantification of phospho-eIF4E (S209) and total eIF4E protein bands with reference to actin control bands (for each CGP57380 concentration) and untreated control using ImageJ Gel Analysis program. (E) Viral protein expression levels upon CGP57380 treatment. EV71-infected RD cells were treated with CGP57380 and cell lysates were harvested for Western blotting at 8h post-treatment. Constant VP0 and VP2 viral protein expression was observed with increasing concentration of the Mnk1 inhibitor. (F) Band intensities of VP0 and VP2 upon CGP57380 treatment. The band intensities representing VP0 and VP2 protein expression level were quantitated with reference to actin control bands (for each concentration) and 1.0% DMSO control using ImageJ Gel Analysis program. (G) Cell viability of CGP57380-treated cells and untreated control cells were measured using alamarBlue assay at 12h post-treatment. Values obtained were normalised against 1.0% DMSO control. Virus titres in the supernatant of cells (denoted by bars) treated with varying concentrations of CGP57380 post-adsorption were analysed via viral plaque assay. Error bars represent standard deviation (SD) of triplicate data. Statistical analyses were performed using one-way ANOVA and Dunnett’s test (Graphpad software) against untreated control *P* > 0.05 (n = 3) versus 1.0% DMSO control (H) RD cells were subjected to infection with EV71 and post-treated with CGP57380 (Mnk1 inhibitor) for 8h. CGP57380-treated cells were fixed and the subcellular localisation of hnRNP A1 (red) was investigated by indirect immunofluorescence assay. Immunofluorescence detection of double-stranded RNA (dsRNA, green) with the nuclei stained with DAPI (blue) was shown to indicate EV71 infection. The images were taken at 100X magnification. Colocalisation quantification was based on the MOC using WCIF ImageJ software and represented as percent colocalisation at the respective drug concentrations. Error bars represent the standard deviation of duplicate data.
(PPTX)

## References

[1] Ho M, Chen ER, Hsu KH, Twu SJ, Chen KT, et al (1999) An epidemic of enterovirus 71 infection in Taiwan. New Engl J Med 341: 929–935. 10498487 10.1056/NEJM199909233411301

[2] Schmidt NJ, Lennette EH, Ho HH (1974) An apparently new enterovirus isolated from patients with disease of the central nervous system. J Infect Dis 129:304–309. 4361245 10.1093/infdis/129.3.304

[3] Cardosa MJ, Krishnan S, Tio PH., Perera D, Wong SC (1999) Isolation of subgenus B adenovirus during a fatal outbreak of enterovirus 71-associated hand, foot, and mouth disease in Sibu, Sarawak. Lancet 354: 987–991. 10501361 10.1016/S0140-6736(98)11032-2

[4] Chan LG, Parashar UD, Lye MS, Ong FG, Zaki SR, et al (2000) Deaths of children during an outbreak of hand, foot, and mouth disease in sarawak, malaysia: clinical and pathological characteristics of the disease. For the Outbreak Study Group. Clinical Infectious Diseases 31: 678–683. 11017815 10.1086/314032

[5] Chan KP, Goh KT, Chong CY, Teo ES, Lau G, et al (2003) Epidemic hand, foot and mouth disease caused by human enterovirus 71, Singapore. Emerg Infect Dis 9: 78–85 12533285 10.3201/eid1301.020112 PMC2873753

[6] Wang JR, Tuan YC, Tsai HP, Yan JJ, Liu CC, et al (2002) Change of major genotype of enterovirus 71 in outbreaks of hand-foot-and mouth disease in Taiwan between 1998 and 2000. J Clin Microbiol 40: 10–15. 11773085 10.1128/JCM.40.1.10-15.2002 PMC120096

[7] Solomon T, Lewthwaite P, Cardosa MJ, McMinn P, Ooi MH (2010) Enterovirus 71—An Emerging Virus With Pandemic Potential. Lancet Infect Dis 10: 778–90. 10.1016/S1473-3099(10)70194-8 20961813

[8] Wu KX, Ng MML, Chu JJH (2010) Developments towards antiviral therapies against enterovirus 71. Drug Discov Today 15(23–24): 1041–1051. 10.1016/j.drudis.2008.12.006 20974282 PMC7108380

[9] Yang Y, Zhang L, Fan X, Qin C, Liu J (2012) Antiviral effect of geraniin on human enterovirus 71 in vitro and in vivo. Bioorg Med Chem Lett 22(6): 2209–2211 10.1016/j.bmcl.2012.01.102 22342145

[10] Smyth MS, Martin JH (2002). Picornavirus uncoating. Mol Pathol 55(4): 214–219. 12147709 10.1136/mp.55.4.214 PMC1187181

[11] McMinn PC (2002) An overview of the evolution of enterovirus 71 and its clinical and public health significance. FEMS Microbiol Rev 26(1): 91–107. 12007645 10.1111/j.1574-6976.2002.tb00601.x

[12] Bedard KM, Semler BL (2004) Regulation of picornavirus gene expression. Microbes Infect 6: 702–713. 15158778 10.1016/j.micinf.2004.03.001

[13] Wimmer E, Nomoto A (1993) Molecular biology and cell-free synthesis of poliovirus. Biologicals 21:349–356. 8024750 10.1006/biol.1993.1095

[14] Shih SR, Stollar V, Li ML (2011) Host factors in enterovirus 71 replication. J Virol 85(19): 9658–9666. 10.1128/JVI.05063-11 21715481 PMC3196451

[15] Back SH, Kim YK, Kim WJ, Cho S, Oh HR, et al (2002) Translation of polioviral mRNA is inhibited by cleavage of polypyrimidine tract-binding proteins executed by polioviral 3C(pro). J Virol 76: 2529–2542. 11836431 10.1128/jvi.76.5.2529-2542.2002 PMC135932

[16] Florez PM, Sessions OM, Wagner EJ, Gromeier M, Garcia-Blanco MA (2005) The polypyrimidine tract binding protein is required for efficient picornavirus gene expression and propagation. J Virol 79: 6172–6179 15858002 10.1128/JVI.79.10.6172-6179.2005 PMC1091667

[17] Hellen CU, Witherell GW, Schmid M, Shin SH, Pestova TV, et al (1993) A cytoplasmic 57-kDa protein that is required for translation of picornavirus RNA by internal ribosomal entry is identical to the nuclear pyrimidine tract-binding protein. Proc Natl Acad Sci U S A 90: 7642–7646 8395052 10.1073/pnas.90.16.7642 PMC47198

[18] Blyn LB, Swiderek KM, Richards O, Stahl DC, Semler BL, et al (1996) Poly(rC) binding protein 2 binds to stem-loop IV of the poliovirus RNA 5′ noncoding region: identification by automated liquid chromatography-tandem mass spectrometry. Proc Natl Acad Sci USA 93:11115–11120 8855318 10.1073/pnas.93.20.11115 PMC38293

[19] Huang PN, Lin JY, Locker N, Kung YA, Hung CT, et al (2011) Far upstream element binding protein 1 binds the internal ribosomal entry site of enterovirus 71 and enhances viral translation and viral growth. Nucleic Acids Res 39:9633–9648 10.1093/nar/gkr682 21880596 PMC3239202

[20] Lin JY, Shih SR, Pan M, Li C, Lue CF, et al (2009) hnRNP A1 interacts with the 5′ untranslated regions of enterovirus 71 and Sindbis virus RNA and is required for viral replication. J Virol. 83: 6106–6114. 10.1128/JVI.02476-08 19339352 PMC2687368

[21] Kyriakis JM (1999) Signaling by the Germinal Center Kinase Family of Protein Kinases. J Biol Chem 274(9): 5259–5262. 10026130 10.1074/jbc.274.9.5259

[22] Hu Y, Leo C, Yu S, Huang BC, Wang H, et al (2004) Identification and functional characterization of a novel human misshapen/Nck interacting kinase-related kinase, hMINK beta. J Biol Chem 279: 54387–54397. 15469942 10.1074/jbc.M404497200

[23] Dan I, Watanabe NM, Kobayashi T, Yamashita-Suzuki K, Fukagaya Y, et al (2000) Molecular cloning of MINK, a novel member of mammalian GCK family kinases, which is up-regulated during postnatal mouse cerebral development. FEBS Lett 469(1): 19–23. 10708748 10.1016/s0014-5793(00)01247-3

[24] Garrington TP, Johnson GL (1999) Organization and regulation of mitogen-activated protein kinase signaling pathways. Curr Opin in Cell Biol 11(2): 211–218. 10209154 10.1016/s0955-0674(99)80028-3

[25] Hirasawa K, Kim A, Han HS, Han J, Jun HS (2003) Effect of p38 Mitogen-Activated Protein Kinase on the Replication of Encephalomyocarditis Virus. J Virol 77(10): 5649–5656. 12719557 10.1128/JVI.77.10.5649-5656.2003 PMC154047

[26] Hussain KM, Leong KL, Ng MM, Chu JJ (2011) The essential role of clathrin-mediated endocytosis in the infectious entry of human enterovirus 71. J Biol Chem 286: 309–321. 10.1074/jbc.M110.168468 20956521 PMC3012988

[27] Tomida J, Kitao H, Kinoshita E, Takata M (2008) Detection of phosphorylation on large proteins by western blotting using Phos-tag containing gel. Protocol exchange. Available: www.nature.com/protocolexchange.protocols/501. Accessed 26 November 2013.

[28] Yamayoshi S, Yamashita Y, Li J, Hanagata N, Minowa T, et al (2009) Scavenger receptor B2 is a cellular receptor for Enterovirus 71. Nat Med 15(7):798–801. 10.1038/nm.1992 19543282

[29] Hasan NM, Adams GE, Joiner MC (1999) Effect of serum starvation on expression and phosphorylation of PKC-α and p53 in V79 cells: Implications for cell death. Int J Cancer 80(3): 400–405. 9935181 10.1002/(sici)1097-0215(19990129)80:3<400::aid-ijc11>3.0.co;2-u

[30] Chakravortty D, Kato Y, Sugiyama T, Koide N, Mu MM, et al (2001) Inhibition of p38 Mitogen-Activated Protein Kinase Augments Lipopolysaccharide-Induced Cell Proliferation in CD14-Expressing Chinese Hamster Ovary Cells. Infect Immun 69: 931–936 11159988 10.1128/IAI.69.2.931-936.2001 PMC97972

[31] Si X., McManus BM, Zhang J, Yuan J, Cheung C, Esfandiarei M, Suarez A, Morgan A, Luo H (2005) Pyrrolidine dithiocarbamate reduces coxsackievirus B3 replication through inhibition of the ubiquitin-proteasome pathway. J. Virol 79:8014–8023. 15956547 10.1128/JVI.79.13.8014-8023.2005 PMC1143712

[32] Bek EJ, McMinn PC (2010) Recent advances in research on human enterovirus 71. Future Virol 5(4): 453–468.

[33] Chen YJ, Ng SJ, Hsu JTA, Horng JT, Yang HM Shih, et al (2008). Amantadine as a regulator of internal ribosome entry site. Acta Pharmacol Sin 29:1327–1333. 10.1111/j.1745-7254.2008.00876.x 18954527

[34] Goetz C, Everson RG, Zhang LC, Gromeier M (2010) MAPK signal-integrating kinase controls cap-independent translation and cell type-specific cytotoxicity of an oncolytic poliovirus.Mol Ther 18: 1937–1946. 10.1038/mt.2010.145 20648000 PMC2990508

[35] van der Houven van Oordt W, Diaz-Meco MT, Lozano J, Krainer AR, Moscat J, et al (2000) The MKK(3/6)-p38-signaling cascade alters the subcellular distribution of hnRNP A1 and modulates alternative splicing regulation. J Cell Biol 149: 307–316 10769024 10.1083/jcb.149.2.307 PMC2175157

[36] Manders EEM, Verbeek FJ, Aten JA (1993) Measurement of colocalisation of objects in dual-colour confocal images. J. Microsc 169: 375–382. 10.1111/j.1365-2818.1993.tb03313.x 33930978

[37] Ziaei S, Shimada N, Kucharavy H, Hubbard K (2012) MNK1 expression increases during cellular senescence and modulates the subcellular localization of hnRNP A1. Exp Cell Res 10; 318(5):500–8 10.1016/j.yexcr.2011.12.015 22227431 PMC3288735

[38] Caignard G, Guerbois M, Labernardiere JL, Jacob Y, Jones LM et al (2007) Measles virus V protein blocks Jak1-mediated phosphorylation of STAT1 to escape IFN-alpha/beta signalling. Virology 368: 351–362. 17686504 10.1016/j.virol.2007.06.037

[39] Hsu MJ, Wu CY, Chiang HH, Lai YL, Hung SL (2010) PI3K/Akt signaling mediated apoptosis blockage and viral gene expression in oral epithelial cells during herpes simplex virus infection. Virus Res 153: 36–43. 10.1016/j.virusres.2010.07.002 20620179

[40] Nemerow GR, Stewart PL (1999) Role of alpha(v) integrins in adenovirus cell entry and gene delivery. Microbiol Mol Biol R 63: 725–734. 10477314 10.1128/mmbr.63.3.725-734.1999 PMC103752

[41] Barber SA, Bruett L, Douglass BR, Herbst DS, Zink MC, et al (2002) Visna virus-induced activation of MAPK is required for virus replication and correlates with virus-induced neuropathology. J Virol 76: 817–828 11752171 10.1128/JVI.76.2.817-828.2002 PMC136850

[42] Tung WH, Hsieh HL, Yang CM (2010) Enterovirus 71 induces COX-2 expression via MAPKs, NF-kappaB, and AP-1 in SK-N-SH cells: Role of PGE(2) in viral replication. Cell Signal 22: 234–246. 10.1016/j.cellsig.2009.09.018 19800403

[43] Wong WR, Chen YY, Yang SM, Chen YL, Horng JT (2005) Phosphorylation of PI3K/Akt and MAPK/ERK in an early entry step of enterovirus 71. Life Sci 78: 82–90. 16150462 10.1016/j.lfs.2005.04.076 PMC7094582

[44] Johnston JB, Barrett JW, Chang W, Chung CS, Zeng W, et al (2003) Role of the Serine-Threonine Kinase PAK-1 in Myxoma Virus Replication. J Virol 77, 5877–5888. 12719581 10.1128/JVI.77.10.5877-5888.2003 PMC154029

[45] Carreno S, Engqvist-Goldstein AE, Zhang CX, McDonald KL, Drubin DG (2004) Actin dynamics coupled to clathrin-coated vesicle formation at the trans-Golgi network. J Cell Biol 165: 781–788. 15210728 10.1083/jcb.200403120 PMC2172402

[46] Merrifield CJ, Feldman ME, Wan L, Almers W (2002) Imaging actin and dynamin recruitment during invagination of single clathrin-coated pits. Nat Cell Biol 4: 691–698. 12198492 10.1038/ncb837

[47] Liu CC, Chou AH, Lien SP, Lin HY, Liu SJ, et al (2011) Identification and characterization of a cross-neutralization epitope of EV71. Vaccine 29: 4362–73. 10.1016/j.vaccine.2011.04.010 21501643

[48] Liu CC, Guo MS, Lin FHY, Hsiao KN, Chang KHW, et al (2011) Purification and characterization of EV71 viral particles produced from Vero cell grown in a serum-free microcarrier bioreactor system. PloS ONE 10.1371/journal.pone.0020005 PMC3094384 21603631

[49] Nicke B, Bastien J, Khanna SJ, Warne PH, Cowling V, et al (2005) Involvement of MINK, a Ste20 family kinase, in Ras oncogene-induced growth arrest in human ovarian surface epithelial cells. Molec Cell 20(5): 673–685. 16337592 10.1016/j.molcel.2005.10.038

[50] Banerjee S, Narayanan K, Mizutani T, Makino S (2002) Murine Coronavirus Replication-Induced p38 Mitogen-Activated Protein Kinase Activation Promotes Interleukin-6 Production and Virus Replication in Cultured Cells. J Virol 76: 5937–5948. 12021326 10.1128/JVI.76.12.5937-5948.2002 PMC136219

[51] Chang WW, Su IJ, Chang WT, Hwang W, Lei HY (2008) Suppression of p38 mitogen-activated protein kinase inhibits hepatitis B virus replication in human hepatoma cell: the antiviral role of nitric oxide. J Viral Hepat 15(7):490–497. 10.1111/j.1365-2893.2007.00968.x 18221299

[52] Thompson SR, Sarnow P (2003) Enterovirus 71 contains a type I IRES element that functions when eukaryotic initiation factor eIF4G is cleaved. Virology 315(1): 259–266. 14592777 10.1016/s0042-6822(03)00544-0

[53] Ho BC, Yu SL, Chen JJ, Chang SY, Yan BS, et al (2011) Enterovirus-induced miR-141 contributes to shutoff of host protein translation by targeting the translation initiation factor eIF4E. Cell Host Microbe 9: 58–69. 10.1016/j.chom.2010.12.001 21238947

[54] Welnowska E, Sanz MA, Redondo N, Carrasco L (2011) Translation of Viral mRNA without Active eIF2: The Case of Picornaviruses. PLoS ONE 6(7): e22230 10.1371/journal.pone.0022230 21779397 PMC3136507

[55] Fernandez J, Yaman I, Sarnow P, Snider MD, Hatzoglou M (2002) Regulation of internal ribosomal entry site-mediated translation by phosphorylation of the translation initiation factor eIF2alpha. J Biol Chem 277: 19198–19205. 11877448 10.1074/jbc.M201052200

[56] Hershey JW (1989) Protein phosphorylation controls translation rates. J Biol Chem 264: 20823–20826. 2687263

[57] Pisarev AV, Shirokikh NE, Hellen CU (2005) Translation initiation by factor-independent binding of eukaryotic ribosomes to internal ribosomal entry sites. CR Biol 328: 589–605. 15992743 10.1016/j.crvi.2005.02.004

[58] Robert F, Kapp LD, Khan SN, Acker MG, Kolitz S, et al (2006) Initiation of protein synthesis by hepatitis C virus is refractory to reduced eIF2.GTP.Met-tRNA(i) (Met) ternary complex availability. Mol Biol Cell 17: 4632–4644 16928960 10.1091/mbc.E06-06-0478 PMC1635388

[59] Black TL, Safer B, Hovanessian A, Katze MG (1989) The cellular 68,000-Mr protein kinase is highly autophosphorylated and activated yet significantly degraded during poliovirus infection: implications for translational regulation. J Virol 63: 2244–2251. 2539516 10.1128/jvi.63.5.2244-2251.1989 PMC250642

[60] Spriggs KA, Bushell M, Mitchell SA, Willis AE (2005) Internal ribosome entry segment-mediated translation during apoptosis: the role of IRES-trans-acting factors. Cell death and differentiation 12: 585–591. 15900315 10.1038/sj.cdd.4401642

[61] Cammas A, Pileur F, Bonnal S, Lewis SM, Leveque N, et al (2007) Cytoplasmic Relocalization of Heterogeneous Nuclear Ribonucleoprotein A1 Controls Translation Initiation of Specific mRNAs. Mol Biol Cell 18: 5048–5059. 17898077 10.1091/mbc.E07-06-0603 PMC2096577

[62] Pin˜ol-Roma S, Dreyfuss G (1992) Shuttling of pre-mRNA binding proteins between nucleus and cytoplasm. Nature 355: 730–732. 1371331 10.1038/355730a0

[63] Monette A, Ajamian L, Lopez-Lastra M, Mouland AJ (2009) Human immunodeficiency virus type 1 (HIV-1) induces the cytoplasmic retention of heterogeneous nuclear ribonucleoprotein A1 by disrupting nuclear import: implications for HIV-1 gene expression. J Biol Chem 284: 31350–31362. 10.1074/jbc.M109.048736 19737937 PMC2781532

[64] Nakielny S, Dreyfuss G (1999) Transport of proteins and RNAs in and out of the nucleus. Cell 99: 677–690. 10619422 10.1016/s0092-8674(00)81666-9

[65] Singh S, Poh CL, Chow VT (2002) Complete sequence analyses of enterovirus 71 strains from fatal and non-fatal cases of the hand, foot and mouth disease outbreak in Singapore (2000). Microbiol Immunol 46: 801–808. 12516778 10.1111/j.1348-0421.2002.tb02767.x

[66] Romero JR, Rotbart HA (1995) Sequence analysis of the downstream 5′ nontranslated region of seven echoviruses with different neurovirulence phenotypes. J Virol 69: 1370–1375. 7815524 10.1128/jvi.69.2.1370-1375.1995 PMC188723

[67] Phuektes P, Chua BH, Sanders S, Bek EJ, Kok CC, et al (2011) Mapping genetic determinants of the cell-culture growth phenotype of enterovirus 71. J Gen Virol 92(6): 1380–1390. 10.1099/vir.0.029371-0 21346025 PMC3168283

