# Supplementary material for: The Role of Misshapen NCK-related kinase (MINK), a Novel Ste20 Family Kinase, in the IRES-Mediated Protein Translation of Human Enterovirus 71
Source: PLoS Pathog. 2015 Mar 6;11(3):e1004686. doi: 10.1371/journal.ppat.1004686 (PMC4352056; doi:10.1371/journal.ppat.1004686)
Supplement: S1 Table — (PPTX) [file ppat.1004686.s001.pptx]

## Slide 1
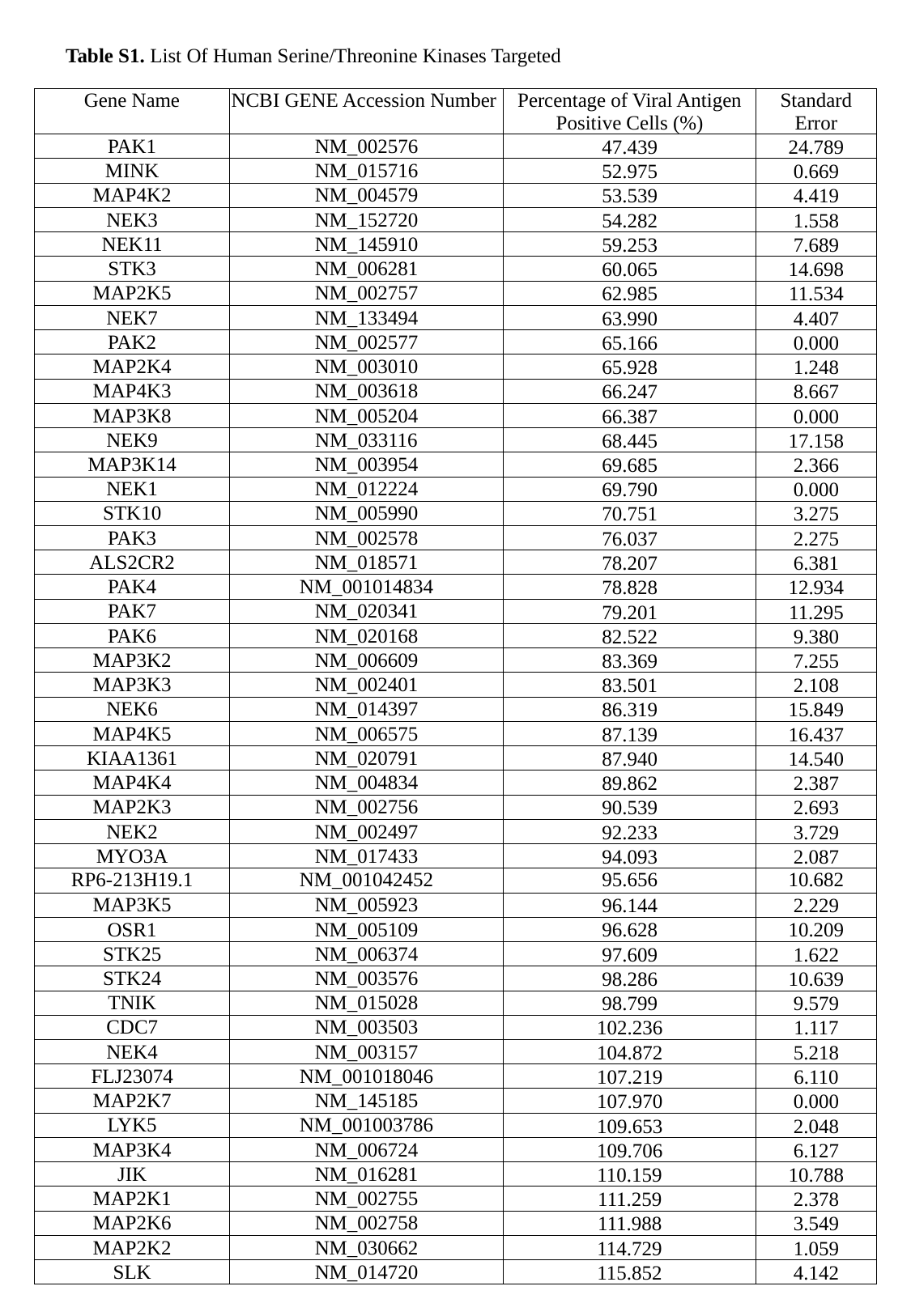

Table S1. List Of Human Serine/Threonine Kinases Targeted
| Gene Name | NCBI GENE Accession Number | Percentage of Viral Antigen Positive Cells (%) | Standard Error |
| --- | --- | --- | --- |
| PAK1 | NM\_002576 | 47.439 | 24.789 |
| MINK | NM\_015716 | 52.975 | 0.669 |
| MAP4K2 | NM\_004579 | 53.539 | 4.419 |
| NEK3 | NM\_152720 | 54.282 | 1.558 |
| NEK11 | NM\_145910 | 59.253 | 7.689 |
| STK3 | NM\_006281 | 60.065 | 14.698 |
| MAP2K5 | NM\_002757 | 62.985 | 11.534 |
| NEK7 | NM\_133494 | 63.990 | 4.407 |
| PAK2 | NM\_002577 | 65.166 | 0.000 |
| MAP2K4 | NM\_003010 | 65.928 | 1.248 |
| MAP4K3 | NM\_003618 | 66.247 | 8.667 |
| MAP3K8 | NM\_005204 | 66.387 | 0.000 |
| NEK9 | NM\_033116 | 68.445 | 17.158 |
| MAP3K14 | NM\_003954 | 69.685 | 2.366 |
| NEK1 | NM\_012224 | 69.790 | 0.000 |
| STK10 | NM\_005990 | 70.751 | 3.275 |
| PAK3 | NM\_002578 | 76.037 | 2.275 |
| ALS2CR2 | NM\_018571 | 78.207 | 6.381 |
| PAK4 | NM\_001014834 | 78.828 | 12.934 |
| PAK7 | NM\_020341 | 79.201 | 11.295 |
| PAK6 | NM\_020168 | 82.522 | 9.380 |
| MAP3K2 | NM\_006609 | 83.369 | 7.255 |
| MAP3K3 | NM\_002401 | 83.501 | 2.108 |
| NEK6 | NM\_014397 | 86.319 | 15.849 |
| MAP4K5 | NM\_006575 | 87.139 | 16.437 |
| KIAA1361 | NM\_020791 | 87.940 | 14.540 |
| MAP4K4 | NM\_004834 | 89.862 | 2.387 |
| MAP2K3 | NM\_002756 | 90.539 | 2.693 |
| NEK2 | NM\_002497 | 92.233 | 3.729 |
| MYO3A | NM\_017433 | 94.093 | 2.087 |
| RP6-213H19.1 | NM\_001042452 | 95.656 | 10.682 |
| MAP3K5 | NM\_005923 | 96.144 | 2.229 |
| OSR1 | NM\_005109 | 96.628 | 10.209 |
| STK25 | NM\_006374 | 97.609 | 1.622 |
| STK24 | NM\_003576 | 98.286 | 10.639 |
| TNIK | NM\_015028 | 98.799 | 9.579 |
| CDC7 | NM\_003503 | 102.236 | 1.117 |
| NEK4 | NM\_003157 | 104.872 | 5.218 |
| FLJ23074 | NM\_001018046 | 107.219 | 6.110 |
| MAP2K7 | NM\_145185 | 107.970 | 0.000 |
| LYK5 | NM\_001003786 | 109.653 | 2.048 |
| MAP3K4 | NM\_006724 | 109.706 | 6.127 |
| JIK | NM\_016281 | 110.159 | 10.788 |
| MAP2K1 | NM\_002755 | 111.259 | 2.378 |
| MAP2K6 | NM\_002758 | 111.988 | 3.549 |
| MAP2K2 | NM\_030662 | 114.729 | 1.059 |
| SLK | NM\_014720 | 115.852 | 4.142 |
