# Supplementary figures and images for: The Role of Misshapen NCK-related kinase (MINK), a Novel Ste20 Family Kinase, in the IRES-Mediated Protein Translation of Human Enterovirus 71
Source: PLoS Pathog. 2015 Mar 6;11(3):e1004686. doi: 10.1371/journal.ppat.1004686 (PMC4352056; doi:10.1371/journal.ppat.1004686)

## Slide 1
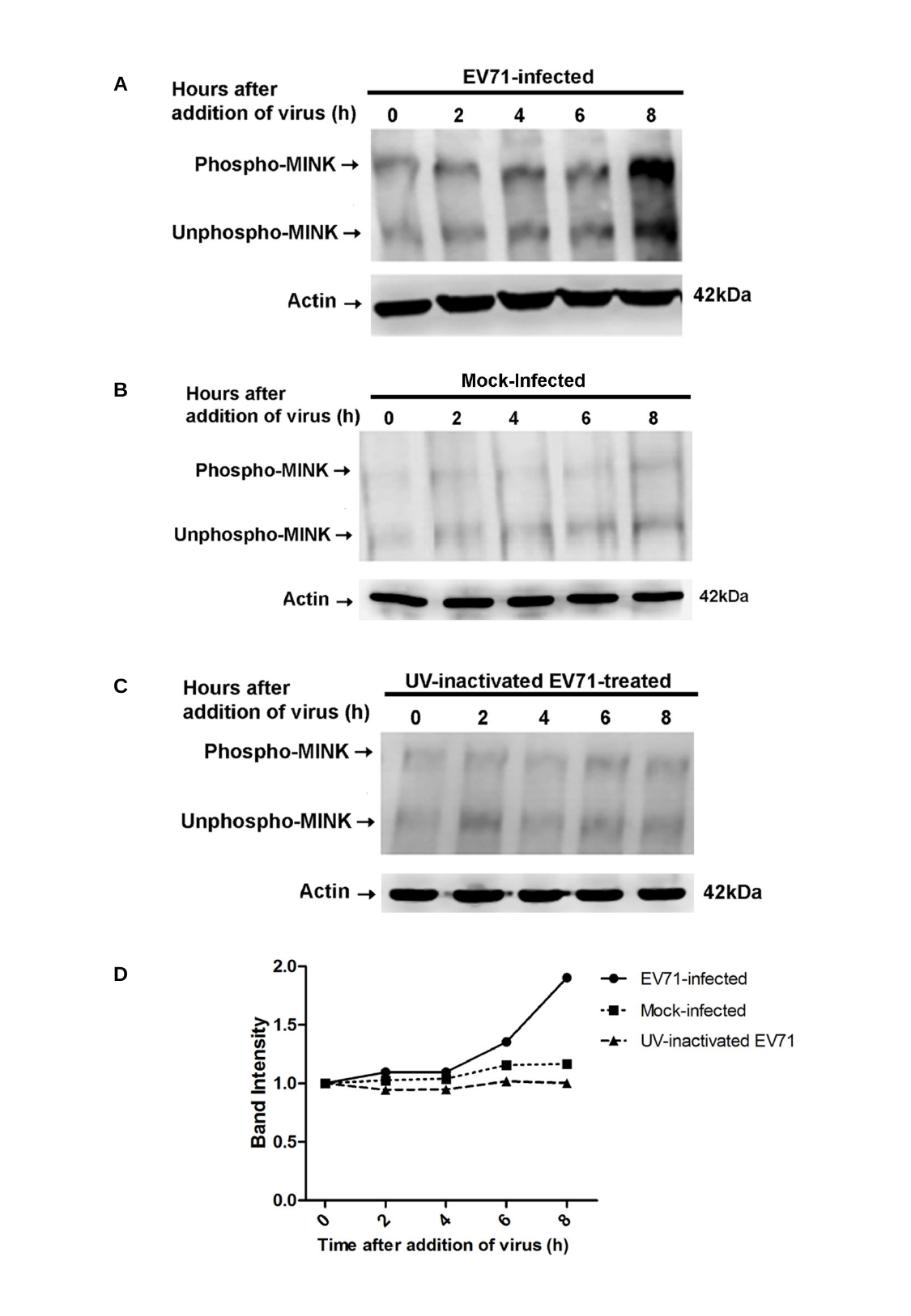

A
B
C
D

Supplement: S1 Fig — (A) A sharp increase in MINK phosphorylation level was observed at 8h after the addition of virus in EV71-infected samples. (B) Low constant levels of phospho-MINK observed in mock-infected cells across the 8h time course. (C) Exposure to UV-inactivated EV71 showed a similar trend of phospho-MINK levels in mock-infected samples. (D) Quantification of phospho-MINK protein bands with reference to actin control bands (for each time-point) and 0h using ImageJ Gel Analysis program. (PPTX) [file ppat.1004686.s002.pptx]

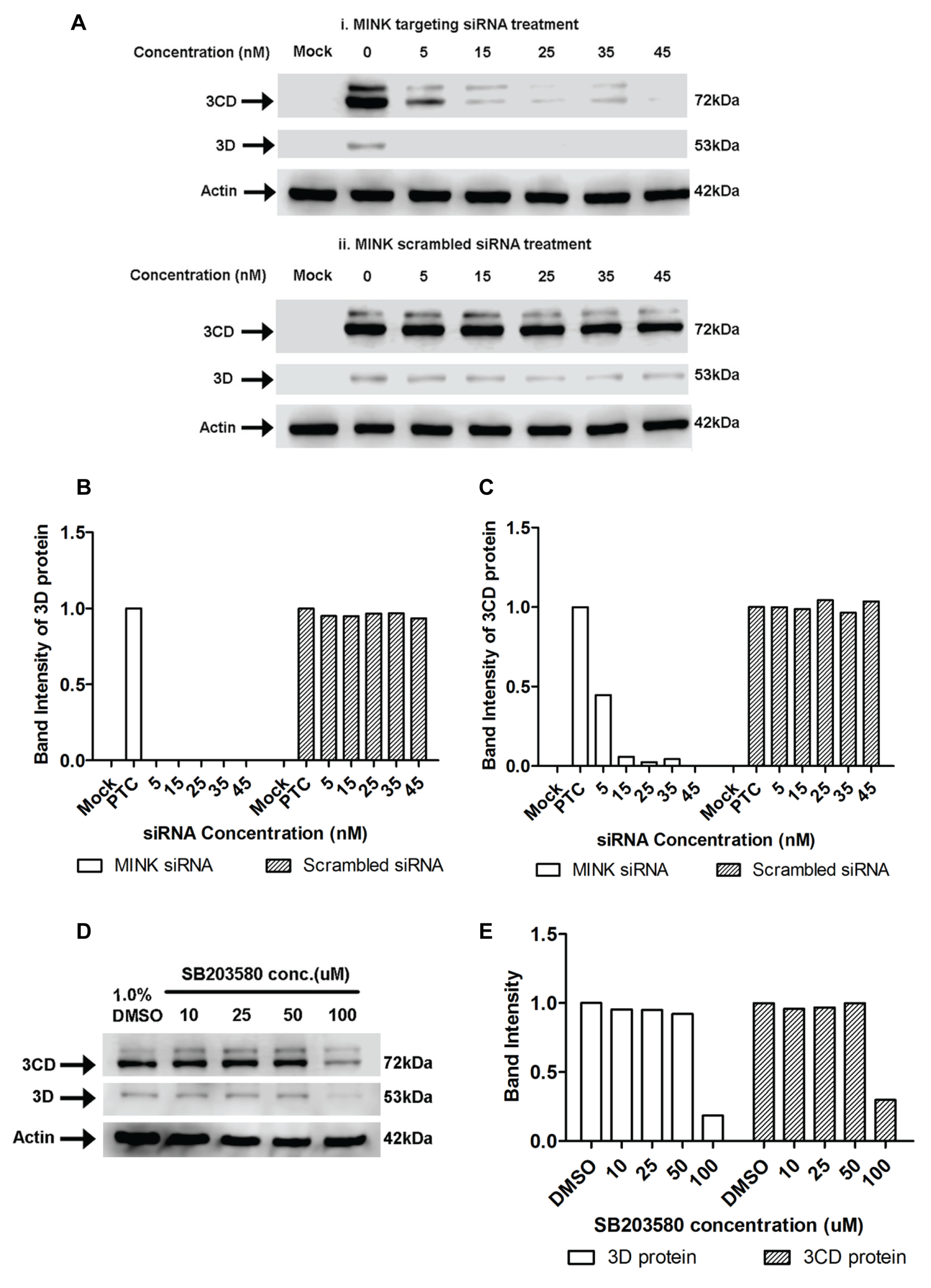

Supplement: S2 Fig — (A) Viral 3D protein expression levels upon the silencing of MINK. Viral protein expression was observed to decrease with increasing concentration of siRNA targeting MINK. (B) Band intensities of 3D protein upon siRNA knockdown of MINK. The band intensities representing 3D protein expression level were quantitated with reference to actin control bands (for each siRNA concentration) and 0nM using ImageJ Gel Analysis program. (C) Band intensities of 3CD protein upon siRNA knockdown of MINK. The band intensities representing 3CD protein expression level were quantitated with reference to actin control bands (for each siRNA concentration) and 0nM using ImageJ Gel Analysis program. (D) EV71 3D protein expression levels upon SB203580 treatment. EV71-infected RD cells were treated with SB203580 and cell lysates were harvested for Western blotting at 8h post-treatment. 3CD and 3D viral protein expression was observed to decrease with increasing concentration of the p38 MAPK inhibitor. (E) Band intensities of 3D and 3CD upon SB203580 treatment. The band intensities representing 3D and 3CD expression level were quantitated with reference to actin control bands (for each concentration) and 1.0% DMSO control using ImageJ Gel Analysis program. (TIF) [file ppat.1004686.s003.tif]

## Slide 1
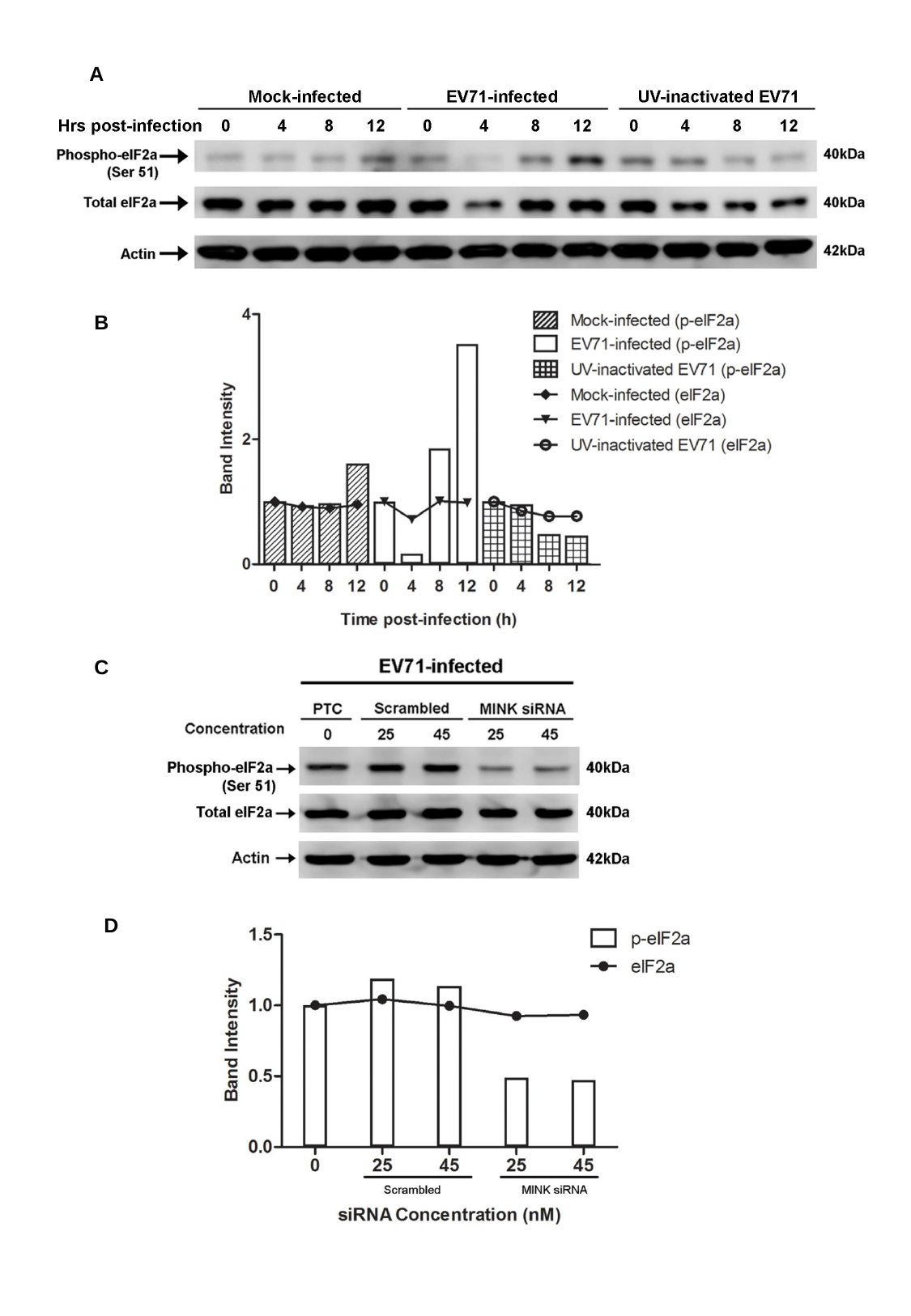

A
B
C
D

Supplement: S3 Fig — (A) A sharp increase in eIF2α phosphorylation level was observed from 8h onwards after the addition of virus in EV71-infected samples. Low constant levels of phospho-eIF2α observed in mock-infected cells with slight increase at 12h. Exposure to UV-inactivated EV71 showed low basal phospho-eIF2α level across the 12h time course. (B) Quantification of phospho-eIF2α and total eIF2α protein bands with reference to actin control bands (for each time-point) and 0hpi using ImageJ Gel Analysis program. (C) Western blot analysis of the phosphorylation levels of eIF2α at 8hpi in infected cells pre-treated with either scrambled or MINK siRNA. β-actin was included as a loading control. (D) Quantification of phospho-eIF2α protein bands with reference to actin control bands (for each concentration) and PTC using ImageJ Gel Analysis program. (PPTX) [file ppat.1004686.s004.pptx]

## Slide 1
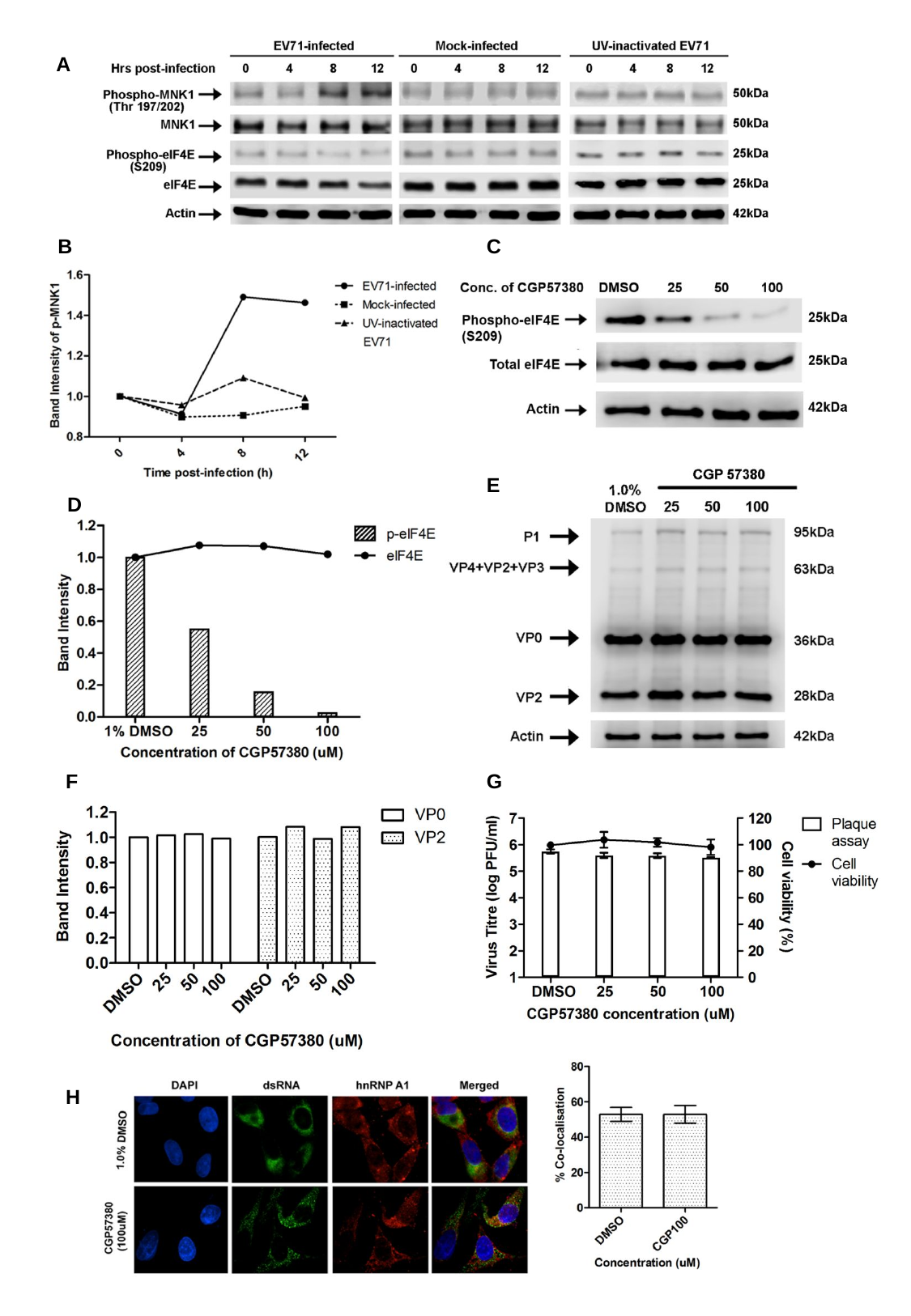

A
B
C
E
D
F
G
H

Supplement: S4 Fig — (A) Western blot analyses of the activation profile of Mnk1 and eIF4E in cells subjected to the three treatments (EV71 infection, mock infection and UV-inactivated EV71). β-actin was included as a loading control. EV71 infection induced the phosphorylation of Mnk1 but downregulated eIF4E protein expression. (B) Quantification of phospho-Mnk1 (Thr197/202) protein bands with reference to actin control bands (for each time-point) and 0hpi using ImageJ Gel Analysis program. (C) Mock-infected RD cells were treated with CGP57380 at different concentrations (25, 50 and 100μM) or 1.0% DMSO (negative control) and cell lysates were harvested for Western blotting at 8h post-treatment. β-actin was included as a loading control. (D) Quantification of phospho-eIF4E (S209) and total eIF4E protein bands with reference to actin control bands (for each CGP57380 concentration) and untreated control using ImageJ Gel Analysis program. (E) Viral protein expression levels upon CGP57380 treatment. EV71-infected RD cells were treated with CGP57380 and cell lysates were harvested for Western blotting at 8h post-treatment. Constant VP0 and VP2 viral protein expression was observed with increasing concentration of the Mnk1 inhibitor. (F) Band intensities of VP0 and VP2 upon CGP57380 treatment. The band intensities representing VP0 and VP2 protein expression level were quantitated with reference to actin control bands (for each concentration) and 1.0% DMSO control using ImageJ Gel Analysis program. (G) Cell viability of CGP57380-treated cells and untreated control cells were measured using alamarBlue assay at 12h post-treatment. Values obtained were normalised against 1.0% DMSO control. Virus titres in the supernatant of cells (denoted by bars) treated with varying concentrations of CGP57380 post-adsorption were analysed via viral plaque assay. Error bars represent standard deviation (SD) of triplicate data. Statistical analyses were performed using one-way ANOVA and Dunnett’s test (Graphpad so [file ppat.1004686.s005.pptx]
